# Mobilization of Iron Stored in Bacterioferritin Is Required for Metabolic Homeostasis in *Pseudomonas aeruginosa*

**DOI:** 10.3390/pathogens9120980

**Published:** 2020-11-24

**Authors:** Achala N. D. Punchi Hewage, Leo Fontenot, Jessie Guidry, Thomas Weldeghiorghis, Anil K. Mehta, Fabrizio Donnarumma, Mario Rivera

**Affiliations:** 1 Department of Chemistry, University of Kansas, 2030 Becker Dr., Lawrence, KS 66047, USA; achala@ku.edu; 2 Department of Chemistry, Louisiana State University, 232 Choppin Hall, Baton Rouge, LA 70803, USA; lfont39@lsu.edu (L.F.); thomaskw@lsu.edu (T.W.); fabrizio@lsu.edu (F.D.); 3 Department of Biochemistry and Molecular Biology, Louisiana State University Health Science Center, 1901 Perdido Street, New Orleans, LA 70112, USA; jjguid@lsuhsc.edu; 4 National High Magnetic Field Laboratory, University of Florida, 1149 Newell Drive, Gainesville, FL 32610, USA; anil.mehta@ufl.edu

**Keywords:** iron homeostasis, iron metabolism, sulfur metabolism, *Pseudomonas aeruginosa*, ferritin, bacterioferritin, ferredoxin, proteomics

## Abstract

Iron homeostasis offers a significant bacterial vulnerability because pathogens obtain essential iron from their mammalian hosts, but host-defenses maintain vanishingly low levels of free iron. Although pathogens have evolved mechanisms to procure host-iron, these depend on well-regulated iron homeostasis. To disrupt iron homeostasis, our work has targeted iron mobilization from the iron storage protein bacterioferritin (BfrB) by blocking a required interaction with its cognate ferredoxin partner (Bfd). The blockade of the BfrB–Bfd complex by deletion of the *bfd* gene (Δ*bfd*) causes iron to irreversibly accumulate in BfrB. In this study we used mass spectrometry and NMR spectroscopy to compare the proteomic response and the levels of key intracellular metabolites between wild type (wt) and isogenic Δ*bfd* *P. aeruginosa* strains. We find that the irreversible accumulation of unusable iron in BfrB leads to acute intracellular iron limitation, even if the culture media is iron-sufficient. Importantly, the iron limitation and concomitant iron metabolism dysregulation trigger a cascade of events that lead to broader metabolic homeostasis disruption, which includes sulfur limitation, phenazine-mediated oxidative stress, suboptimal amino acid synthesis and altered carbon metabolism.

## 1. Introduction

*Pseudomonas aeruginosa* is an opportunistic pathogen causative of severe infections, particularly in health care settings and in immunocompromised patients. The emergence and dissemination of multidrug-resistant *Pseudomonas aeruginosa* strains is a significant threat to public health [1,2,3]. The World Health Organization included carbapenem-resistant *P. aeruginosa* in its priority list of pathogens for which new antibiotics are urgently needed [4]. To combat the threat posed by multidrug-resistant organisms, there is an urgent need to discover novel antibiotics and validate new targets in antibacterial research [5,6,7]. A new approach to combat infection currently under investigation is to capitalize on the iron requirement of pathogens for establishing infections [8,9,10,11,12,13,14]. Iron is essential because of its requirements of enzymes mediating respiration, DNA synthesis, amino acid synthesis and many other key metabolic processes [15]. Pathogens must obtain iron from their host, but host nutritional immunity denies the essential nutrient by maintaining very low concentrations of free iron (~10^−20^ M) [15]. The chemical properties of iron also present challenges to invading pathogens in that the reactivity of the ferrous ion (Fe^2+^) toward H_2_O_2_ and O_2_ induces oxidative stress. Consequently, bacterial iron homeostasis (iron acquisition, storage and utilization) is highly regulated to ensure sufficiency for metabolic needs while preventing iron-induced toxicity [16,17].

**Figure 1:**
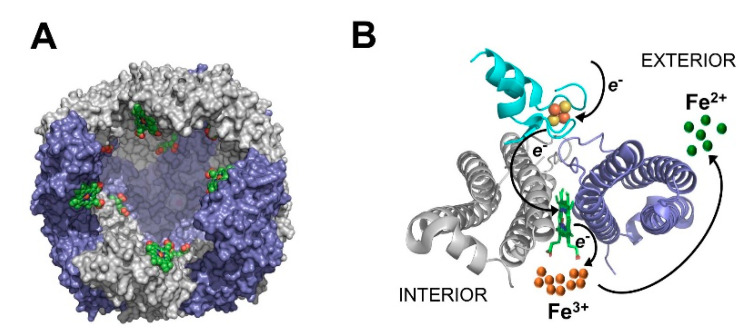
(**A**) Bacterioferritin (BfrB) from *Pseudomonas aeruginosa* (PDB ID 3is7) is a spherical and hollow protein capable of storing up to 3000 Fe^3+^ ions in its interior cavity. Heme molecules (green) are buried under the protein exterior, bound between each two subunits, with the heme propionates exposed to the interior cavity. (**B**) Structure of the BfrB–Bfd complex (PDB ID 4e6k) depicting Bfd (cyan) binding between two BfrB subunits (grey and blue), immediately above a heme molecule. Electron transfer from the [2Fe-2S] cluster in Bfd to the Fe^3+^ in the BfrB interior, which is facilitated by the heme, enables mobilization of Fe^2+^ to the cytosol.

The struggle for iron between host and bacteria has given rise to the idea of exploiting iron metabolism as a potentially viable approach to develop new antibiotics. Approaches that follow this general idea include the development of siderophore conjugates to enhance antibiotic cell penetration [18,19], the sequestration of iron with chelators to deplete intra- and extracellular iron [13,20,21,22,23], the perturbation of heme uptake or heme degradation to deny the pathogens a rich iron source during infections [12,24], and the utilization of Ga^3+^, which is aimed at systemically replacing Fe^3+^ with Ga^3+^ in the active site of enzymes [25,26]. Dysregulation of iron metabolism is also thought to offer a significant vulnerability. To disrupt iron metabolism, our laboratories are studying a new and specific target in *Pseudomonas aeruginosa*, a protein–protein interaction between the iron storage protein bacterioferritin (BfrB) and its physiological partner, the bacterioferritin-associated ferredoxin (Bfd) [27]. Our work has demonstrated that although two ferritin-like proteins (FtnA and BfrB) coexist in *P. aeruginosa* [28], BfrB is the main iron storage protein [16]. BfrB is a spherical molecule assembled from 24 subunits which contains an interior cavity ~80 Å in diameter where up to ~3000 Fe^3+^ ions can be stored (Figure 1A). The BfrB structure also harbors heme molecules sandwiched between two subunits, buried under the external protein surface [27,29]. The mobilization of iron stored in BfrB requires Bfd. The X-ray crystal structure of the BfrB–Bfd complex revealed that up to 12 Bfd molecules can bind at identical sites on the BfrB surface, between two subunits, and immediately above a heme molecule. The function of the heme is to facilitate electron transfer from the [2Fe-2S] cluster in Bfd to the Fe^3+^ stored in the interior cavity, thus facilitating the release of Fe^2+^ to the cytosol (Figure 1B) [30]. 

Studies conducted with a *P. aeruginosa* strain where the *bfd* gene has been deleted (Δ*bfd*), demonstrated that blocking the BfrB–Bfd complex causes the irreversible accumulation of iron in BfrB and low levels of free iron in the cytosol [16]. The same study showed that culturing planktonic Δ*bfd* cells in low iron media results in lower cell density than similar cultures of the wild type (wt) strain. In contrast, when cultured in iron-sufficient media (10 µM), the wt and Δ*bfd* cells grow at the same rate and to the same cell density. In the stationary phase, however, the Δ*bfd* cells secrete several-fold more pyoverdine (an Fe^3+^ siderophore) than the wt cells, suggesting that the blockade of the BfrB–Bfd complex induces iron limitation in the *P. aeruginosa* cells [16]. Additional investigations showed that the Δ*bfd* cells form poorly developed biofilms, even in iron-sufficient conditions, and that the biofilm-embedded cells exhibit cytosolic iron deficiency [14]. Given that *P. aeruginosa* cells require sufficient intracellular iron to establish mature biofilms [31], it was suggested that the irreversible accumulation of iron in BfrB in the Δ*bfd* cells causes iron limitation, even in iron-sufficient conditions [14]. Similarly, challenging planktonic cells with small molecule inhibitors of the BfrB–Bfd complex causes nearly irreversible accumulation of iron in BfrB, a growth phenotype and pyoverdine hyper production [10]. 

Although these observations were interpreted to suggest that the blockade of the BfrB–Bfd complex may result in iron metabolism dysregulation that adversely affects *P. aeruginosa* fitness, the connections between iron metabolism dysregulation and cellular metabolic homeostasis are not yet known [10]. To begin understanding these connections, we interrogated the global cellular response to blocking the BfrB–Bfd complex in *P. aeruginosa* by comparing the proteomic profiles of wt and Δ*bfd* cells cultured in iron-sufficient media. We found that blocking the BfrB–Bfd complex indeed leads to iron metabolism dysregulation and systemic iron limitation. The lesion in iron metabolism triggers a cascade of events, leading to phenazine overproduction and associated redox stress, which impose iron and sulfur limitation, altered carbon metabolism and suboptimal amino acid biosynthesis. 

## 2. Results and Discussion

**Figure 2:**
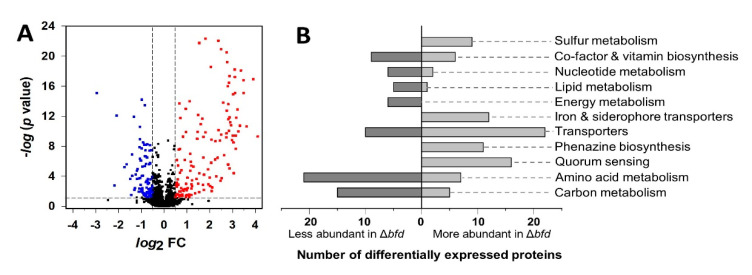
(**A**) Volcano plot illustrating differentially abundant proteins in the Δ*bfd* isogenic mutant relative to the wt strain. The fold change (FC) is defined as the abundance ratio of Δ*bfd*/wt. The non-axial vertical lines designate *log*_2_ FC ± 0.5 and the non-axial horizontal line denotes *p* = 0.05. Points in red represent proteins more abundant in the Δ*bfd* mutant, while points in blue indicate proteins less abundant in the mutant. (**B**) KEGG Orthology functional classification of proteins differentially expressed in wt and Δ*bfd* cells.

Deletion of the *bfd* gene results in the irreversible accumulation of iron in BfrB and concomitant low iron levels in the cytosol [16]. These observations, however, did not indicate whether the low iron levels are confined to the free iron pool in the cytosol, or if iron limitation also affects iron-dependent proteins and the processes in which these proteins participate. Answering this question requires that the experimental approach enable a relatively global assessment of the bacterial cell response to blocking the BfrB–Bfd complex. Consequently, we compared the proteomic profiles of the wild type (wt) *P. aeruginosa* PAO1 and the isogenic Δ*bfd* mutant cultured under identical conditions. As indicated above, both strains grow at the same rate and to a nearly identical cell density when cultured in iron-sufficient media. During the stationary growth phase, however, the Δ*bfd* cells secrete several-fold more pyoverdine than the wt cells because of relatively low cytosolic iron levels [16]. Consequently, we harvested the cells 30 h post inoculation when the Δ*bfd* cells secreted approximately sixfold more pyoverdine than their wt counterparts (Figure S1). To compare the proteomic profiles of the wt and Δ*bfd* mutant cells, we carried out Tandem Mass Tag (TMT) labeling, an isobaric covalent modification of primary amines which enables relative quantification of proteins across multiple samples [32], upstream of the mass spectrometric analysis. This approach allowed us to identify and quantitate 3210 proteins, approximately 54% of the total 5570 proteins in *P. aeruginosa* [33]. Three biological replicates and experimental (three each) replicates were incorporated so that *t*-tests were performed on the 3210 proteins to determine significant differences in protein abundance between wt and Δ*bfd* cells (*p* < 0.05). Proteins exhibiting a Δ*bfd*/wt fold change (FC) ≥ 1.4 (*log*_2_ FC ≥ 0.5) are considered enriched in the Δ*bfd* cells, and proteins with a Δ*bfd*/wt FC ≤ 0.7 (*log*_2_ FC ≤ −0.5) are considered depleted [34]. The results are summarized in a volcano plot (Figure 2) where the segmented vertical lines indicate *log*_2_ FC ± 0.5 and the horizontal segmented line denotes *p* = 0.05. Of the 3210 identified proteins, 296 (~9%) are above or below the cutoff, 159 are enriched (128 *p* < 0.05) and 137 are depleted (96 *p* < 0.05) in Δ*bfd* relative to wt cells. 

The differentially expressed proteins were functionally categorized using the Kyoto Encyclopedia of Genes and Genomes (KEGG) Orthology classification [35] (Figure 2B), the Pseudomonas Genome Database [36], and the UniProt database [37]. This analysis revealed that the inhibition of the BfrB–Bfd complex causes significant changes in the proteins involved in iron metabolism (Table S1). The significant enrichment of proteins involved in iron and heme acquisition in the Δ*bfd* relative to the wt cells is consistent with a strong iron starvation response caused by the irreversible accumulation of iron in BfrB when the BfrB–Bfd complex is inhibited [16]. Accordingly, proteins involved in the biosynthesis of 4-hydroxy-2-alkylquinolines (HAQs) and phenazines are also enriched in the Δ*bfd* cells. Importantly, the data also reveal strong connections between the iron starvation response and carbon metabolism, sulfur metabolism, amino acid biosynthesis, respiration, oxidative stress defense, and cellular transport (Table S1). These findings also indicate that the blockade of the BfrB–Bfd complex is a new approach to establishing intracellular iron starvation without treating the culture media with iron chelators, which can deplete it of other metal ions.

### 2.1. Inhibition of Iron Mobilization from BfrB Elicits a Global Iron Starvation Response

#### 2.1.1. Pyoverdine Biosynthesis

**Figure 3:**
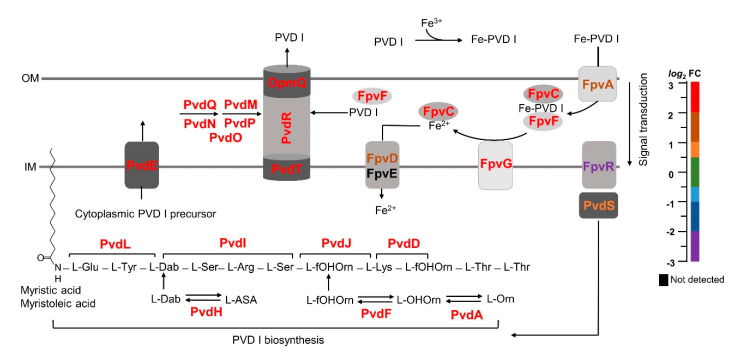
Proteins for pyoverdine (PVD I) assembly and function are enriched in the Δ*bfd* mutant. The schematic shows PVD I assembly, which is initiated in the cytosol, and maturated in the periplasm prior to its secretion to the extracellular milieu. Extracellular PVD I binds Fe^3+^, the Fe^3+^–PVD I complex is internalized to the periplasm where a reductase system facilitates the release of Fe^2+^ and PVD I; the former is transported to the cytosol and the PVD I is recirculated to the extracellular space. The relative abundance (Δ*bfd*/wt) of each of the proteins involved in these processes is color coded according to the *log*_2_FC scale. Outer membrane (OM), inner membrane (IM).

The Δ*bfd* cells overproduce pyoverdine (Figure S1), a siderophore secreted by *P. aeruginosa* to assimilate iron [38]. Pyoverdine molecules are composed of three main parts, a dihydroquinoline chromophore (fluorescent), a strain-specific peptide, and a side chain stemming from the chromophore. *P. aeruginosa* produces three pyoverdines (PVDI-III), each characterized by a different peptide chain [39]; PVDI (PVD hereafter) is the best characterized. PVD biosynthesis, schematically summarized in Figure 3, requires numerous enzymes and non-ribosomal peptide synthetases (NRPSs) [38,40,41]. Inspection of Figure 3 shows that all the enzymes and non-ribosomal peptides involved in the biosynthesis, secretion and import of the iron–PVD complex are strongly enriched in the Δ*bfd* cells, as indicated by the color scale. The PVD backbone begins assembly in the cytosol with the aid of NRPSs, PvdA, PvdD, PvdF, PvdH, PvdI, PvdJ and PvdL. The cytoplasmic (non-fluorescent) PVD precursor bound to a myristic or myristoleic acid chain [42] is thought to be transported into the periplasm by the ABC transporter PvdE [43], which is also highly enriched in the Δ*bfd* cells. The maturation of PVD occurs in the periplasm where PvdQ cleaves the myristic or myristoleic moiety to produce ferribactin, which is maturated by enzymes PvdN, PvdM, PvdO, and PvdP, all significantly enriched in the Δ*bfd* cells. The siderophore is secreted through a tripartite efflux pump consisting of an inner membrane protein (PvdT), periplasmic adaptor protein (PvdR) and outer membrane protein (OpmQ) [41,44,45]. The secreted PVD chelates Fe^3+^ and the complex is taken into the periplasm by FpvA in a TonB-dependent manner [46,47], where it binds to periplasmic proteins FpvC and FpvF. The cytoplasmic membrane protein FpvG is thought to reduce the chelated Fe^3+^, probably aided by proteins FpvH, FpvJ, and FpvK [48]. After its dissociation from PVD, Fe^2+^ binds to FpvC and is transported to the cytoplasm by the ABC transporter FpvDE, and PVD is recycled into the extracellular space via the PvdT–PvdR–OpmQ efflux pump. We also observe that the sigma factor PvdS, required for the expression of pyoverdine, is enriched in the Δ*bfd* cells, whereas the anti-sigma factor FpvR, which is required to inactive PvdS, is strongly attenuated. As such, the proteomic profiles are in excellent agreement with the pyoverdine overproduction of the Δ*bfd* relative to the wt cells. 

#### 2.1.2. Pyochelin Biosynthesis

**Figure 4:**
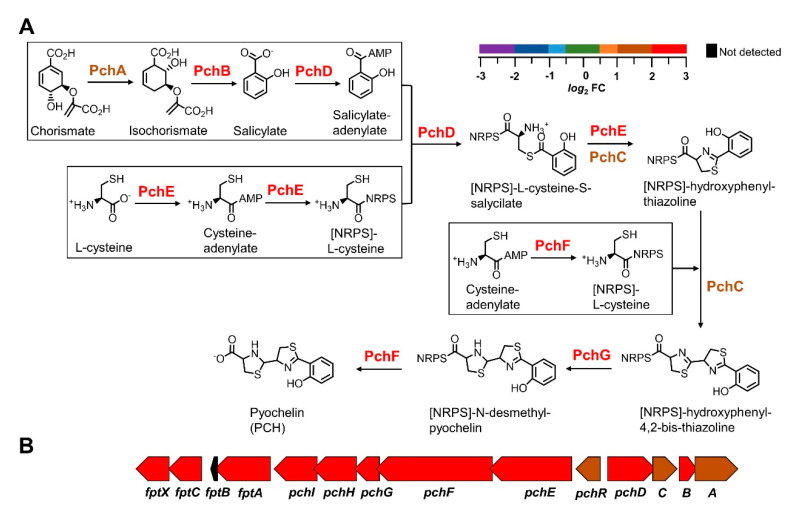
Proteins for the synthesis and function of pyochelin are enriched in the Δ*bfd* cells. (**A**) Schematic depiction of pyochelin (PCH )biosynthesis, which starts with chorismate and requires two equivalents of L-cysteine. The relative abundance (Δ*bfd*/wt) of each of the proteins involved in PCH biosynthesis is color coded according to the *log*_2_FC scale. (**B**) Organization of the genes encoding proteins for PCH biosynthesis and function color coded according to the *log*_2_FC scale.

Pyochelin (PCH) is a lower affinity siderophore relative to pyoverdine, which at neutral pH chelates Fe^3+^ with 2:1 stoichiometry. The biosynthesis of pyochelin involves three NRPS and far fewer proteins than the biosynthesis of pyoverdine. It is schematically depicted in Figure 4A where the relative abundance of proteins in the Δ*bfd* relative to the wt cells is indicated by the color scale. Pyochelin biosynthesis starts with the transformation of chorismate to salicylate by enzymes PchA and PchB [49,50], both enriched in the Δ*bfd* cells. Salicylate is then activated and coupled to a molecule of activated L-cysteine with the aid of enzymes PchD, PchC and PchE to produce hydroxyphenyl-thiazoline [51,52]; PchF catalyzes the incorporation of a second molecule of activated L-cysteine to form the second thiazoline ring, which is reduced to thiazolidine by PchG, before PchF catalyzes the release of pyochelin [53]. The synthesis of PCH takes place in the cytosol, but not much is yet known regarding the proteins involved in its secretion, or the import of iron-bound PCH. In this context, Figure 4B shows the organization of genes involved in pyochelin biosynthesis and function [50], colored with the relative abundance of the corresponding proteins in the proteomics data set. This representation demonstrates that all the proteins involved in pyochelin biosynthesis and function are enriched in the Δ*bfd* cells.

#### 2.1.3. Heme Uptake

**Figure 5:**
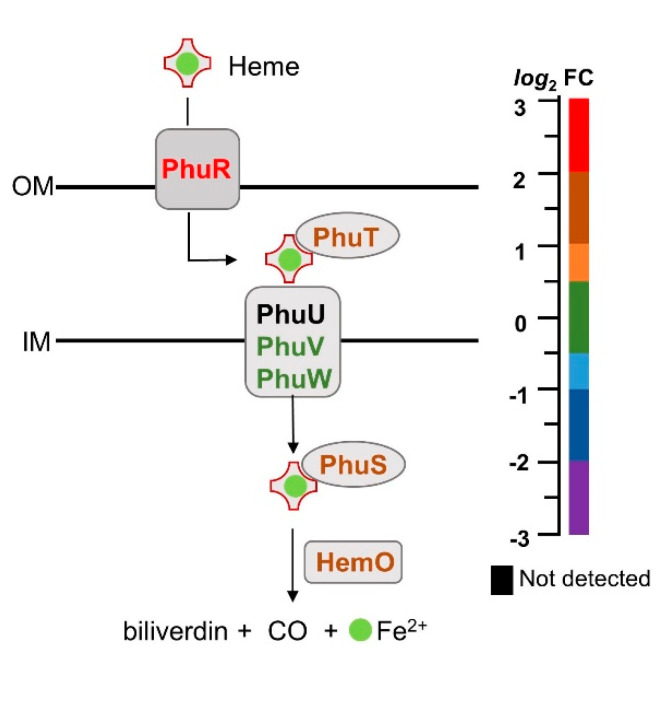
Proteins for heme capture, internalization and release of heme-iron are enriched in the Δ*bfd* cells. The relative abundance (Δ*bfd*/wt) of the proteins involved in these processes is indicated by the color according to the *log*_2_FC scale. Outer membrane (OM), inner membrane (IM).

*P. aeruginosa* utilizes two systems to scavenge host-heme, a hemophore-dependent heme assimilation system (*has*) and a non-hemophore-dependent *Pseudomonas* heme uptake (*phu*) system [54,55]. Heme is extracted from hemoglobin by the TonB-dependent PhuR receptor for transport across the outer membrane. In the periplasmic space heme is shuttled by PhuT to the PhuUVW transporter for internalization into the cytosol, where it is sequestered by PhuS and transferred to the heme oxygenase (HemO) [56], which cleaves the heme macrocycle to release iron, biliverdin-β, biliverdin-δ and carbon monoxide [57]. Consistent with the iron starvation caused by the blockade of the BfrB–Bfd complex, most of the enzymes involved in heme capture, internalization and heme degradation are enriched in the Δ*bfd* cells (Figure 5).

#### 2.1.4. *Pseudomonas* Quinolone Signal

**Figure 6:**
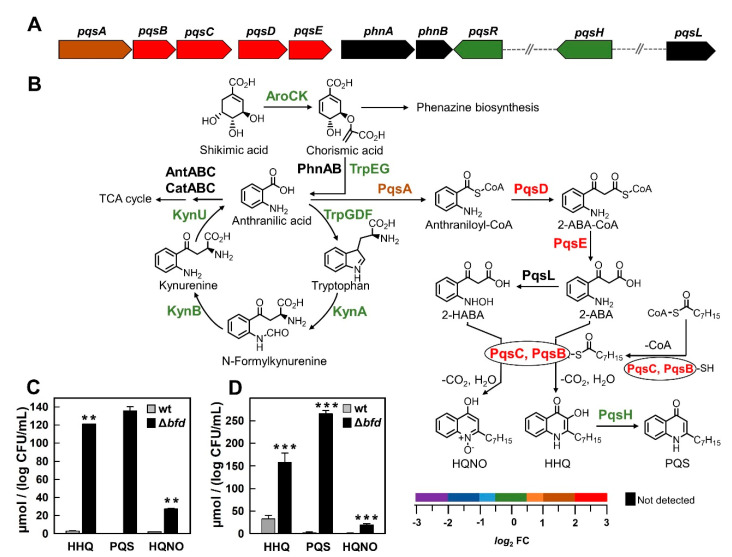
Proteins for the synthesis of AQs are enriched in the Δ*bfd* cells. (**A**) Organization of the genes encoding proteins for AQ biosynthesis, and the *phn*AB genes, color coded according to the *log*_2_FC scale. (**B**) Schematic representation of the biosynthesis of AQs showing the relative abundances (Δ*bfd*/wt) of each of the proteins in the pathway color coded according to the *log*_2_FC scale. The preparation of this scheme was aided significantly by ref [64]. (**C**,**D**) LC-MS-determined levels of HHQ, PQS, and HQNO from cells and from spent media, respectively, obtained from cultures of wt and Δ*bfd* cells; PQS from wt cells was below the detection limit. The amount of AQs has been normalized to *log* CFU/mL. Means and standard deviations from three biological replicates are shown. 2-aminobenzoylacetyl -CoA (2-ABA-CoA), 2-aminobenzoylacetate (2-ABA), 2-hydroxylaminobenzoylacetate (2-HABA). *p* < 0.01 denoted by ** and *p* < 0.001 by *** relative to wt.

The conditions of iron starvation are known to stimulate the biosynthesis of 2-alkyl-4-quinolones (AQs), such as HHQ (2-heptyl-4-hydroxyquinolone), PQS (2-heptyl-3-hydroxy-4-quinolone) and HQNO (2-heptyl-4-hydroxyquinoline-N-oxide). The *pqs* system (Figure 6A) encompasses the *pqs*R gene, the *pqs*ABCDE-*phn*AB operon and the distally located *pqs*H and *pqs*L genes [58]. The enzymes coded by the *pqs* system synthesize HHQ and PQS, which stimulate the expression of the *pqs* operon [59]. Low iron conditions stimulate the biosynthesis of AQs by at least two mechanisms: (i) the iron starvation σ factor PvdS increases transcription of *pqs*R via an iron starvation box [60,61], and (ii) the PrrF sRNAs (PrrF1 and PrrF2), which are expressed under low iron conditions, repress the transcription of genes coding for enzymes that catalyze the degradation of anthranilate (*ant*ABC and *cat*BCA), therefore sparing anthranilate for the biosynthesis of AQs [62,63]. The biosynthetic process, schematically shown in Figure 6B, starts when PqsA converts anthranilate into anthraniloyl-CoA, which is converted to 2-aminobenzoylacetyl-CoA (2-ABA-CoA) by PqsD and then to 2-aminobenzoylacetate (2-ABA) by PqsE. The condensation of 2-ABA with octanoyl-CoA, catalyzed by the PqsBC heterodimer, forms HHQ. Under aerobic conditions HHQ is oxidized to PQS by the enzyme PqsH, whereas the biosynthesis of HQNO, which is also produced from 2-ABA, requires the monooxygenase PqsL and the PqsBC heterodimer [64,65]. 

All the enzymes involved in the conversion of anthranilate to HHQ (*pqs*ABCDE) are enriched in the Δ*bfd* cells. In contrast, the enzyme required to convert 2-ABA to 2-HABA (PqsL) was not detected, and the enzyme required to convert HHQ to PQS (PqsH) is expressed in the Δ*bfd* cells at the same levels as in the wt cells (Figure 6B). In order to understand how this pattern of relative protein expression is manifested in the levels of AQs produced by the wt and Δ*bfd* cells, we used liquid chromatography-mass spectrometry (LC-MS) to quantitate HHQ, PQS and HQNO in the cells and in the cell-free supernatants. These values, normalized to CFU/mL to enable comparisons (Figure 6C,D), demonstrate that the Δ*bfd* cells produce significantly higher levels of AQs than the wt cells, observations that agree with the enrichment of enzymes encoded by the *pqs*ABCDE genes in the mutant. It is also interesting to consider that HHQ and PQS are produced and secreted by the Δ*bfd* cells at relatively similar levels, despite the observation that PqsH is not enriched in Δ*bfd* relative to wt. This is probably because the abundance of HHQ in the Δ*bfd* cells stimulates the activity of PqsH, thus producing relatively high levels of PQS, even if the enzyme is not enriched relative to the wt cells. Along the same vein, the abundance of HHQ in the Δ*bfd* cells probably stimulates the activity of PqsL (not detected) by channeling a fraction of 2-ABA toward 2-HABA, which is converted to HQNO by the PqsBC heterodimer. It is tempting to speculate that the relatively low levels of HQNO relative to HHQ and PQS suggest that the synthesis of HQNO is throttled down, possibly by maintaining low levels of the PqsL enzyme.

Anthranilate, the first committed precursor in the biosynthesis of AQs, can be synthesized from shikimate by AroCK and the anthranilate synthases PhnAB and TrpEG, or via the kynurenine pathway that converts tryptophan into anthranilate [66] (see Figure 6B). We observed no changes in the levels of enzymes involved in the kynurenine pathway or in the enzymes that convert shikimate to anthranilate between wt and Δ*bfd* cells. This is most likely because the peptone in PI media is a rich source of tryptophan, which can be used for anthranilate biosynthesis.

Iron limiting conditions are thought to spare anthranilate for the biosynthesis of AQs by downregulating AntABC and CatABC, the enzymes implicated in the first steps of anthranilate degradation for its incorporation in the TCA cycle [62]. We did not detect AntABC and CatABC in our experiments; nevertheless, the enrichment of enzymes involved in the biosynthesis of AQs, the higher levels of AQs found in the Δ*bfd* cells, and the “normal” production of anthranilate in the Δ*bfd* cells, corroborate that when experiencing iron limitation, *P. aeruginosa* cells indeed utilize anthranilate resources for the biosynthesis of AQs. 

#### 2.1.5. Phenazines

**Figure 7:**
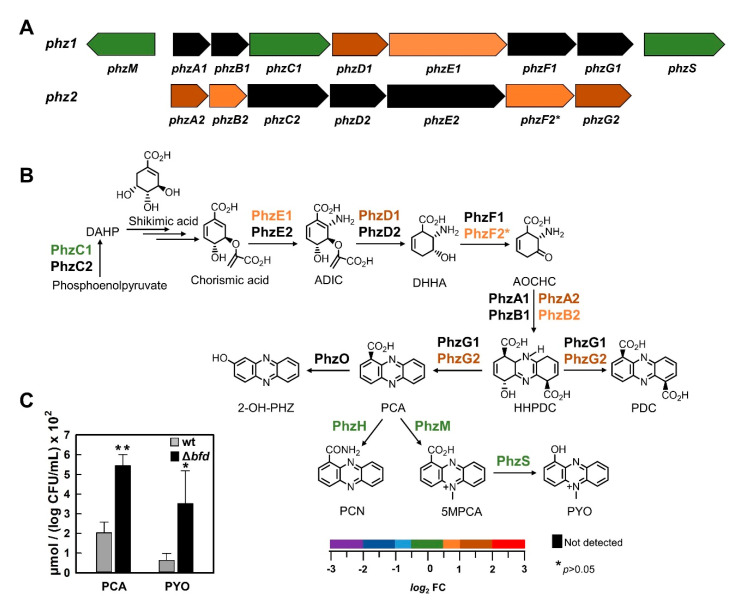
Proteins for the biosynthesis of phenazines are enriched in the Δ*bfd* cells. (**A**) Organization of the genes in the two operons, *phz1* and *phz2*, color coded according to the *log*_2_FC scale. (**B**) Schematic representation for the biosynthesis of phenazines showing the relative abundances (Δ*bfd*/wt) of each of the proteins in the pathway color coded according to the *log*_2_FC scale. The preparation of this scheme was aided significantly by ref. [68]. (**C**) LC-MS-determined levels of PCA in wt and Δ*bfd* cells normalized to *log* CFU/mL. 2-amino-2-desoxyisochorismic acid (ADIC), *trans*- 2,3-dihydro-3-hydroxyanthranilic acid (DHHA), 6-amino-5-oxocyclohex-2-ene-1-carboxylic acid (AOCHC), hexahydrophenazine-1,6-dicarboxylic acid (HHPDC), phenazine-1-carboxylic acid (PCA), 5-methyl-phenazine-1-carboxylic acid (5MPCA), phenazine-1-carboxamide (PCN), pyocyanin (PYO) 2-hydroxy-phenazine (2-OHPHZ). *p* < 0.05 denoted by * and *p* < 0.01 by ** relative to wt.

Although *P. aeruginosa* can produce up to five different phenazine derivatives [67,68], pyocyanin (PYO) is the most studied phenazine in this organism. Phenazine biosynthesis is carried out by enzymes produced from the transcription of two similar operons, *phz*A1B1C1D1E1F1G1 (*phz*1) and *phz*A2B2C2D2E2F2G2 (*phz*2) [67]. HHQ and PQS are known to positively regulate the expression of *phz*1 and *phz*2 [69], and recent studies have shown that the enzyme PqsE also stimulates transcription of the *phz*1 and *phz*2 operons [65]. As such, iron limitation, which stimulates the *pqs*ABCDE-*phn*AB operon (see above), is also expected to stimulate transcription of the *phz* operons via HHQ, PQS and PqsE, all of which are enriched in the Δ*bfd* cells (see Figure 6). In agreement with this idea, the data presented in Figure 7 show that several of the enzymes encoded by genes in the *phz*1 and *phz*2 operons are enriched in the Δ*bfd* cells relative to wt. The enrichment of enzymes coded by both operons is also in agreement with a previous report demonstrating that enzymes from the *phz*1 and *phz*2 operons participate in the biosynthesis of phenazine-1-carboxylic acid (PCA) in planktonic cells [70]. Inspection of Figure 7B shows that in the path converting chorismic acid to PCA, at least one of the required enzymes from the *phz*1 or *phz*2 operons is enriched in the Δ*bfd* cells. In comparison, the enzymes required for the conversion of PCA to 5MPCA (PhzM) and then to PYO (PhzS) exhibit the same levels as in the wt cells. To understand the consequences of this enrichment pattern on the production of phenazines, we analyzed the total levels of PCA and PYO; 5MPCA was not analyzed due to being unstable [71]. The results (Figure 7C) show that the Δ*bfd* cells produce approximately threefold more PCA and PYO than the wt cells, observations that are in excellent agreement with the enrichment of the enzymes required to convert chorismic acid to PCA. The enhanced production of PYO by the Δ*bfd* cells with “normal” levels of PhzS is probably due to its stimulation by the abundance of PCA. The potential consequences of PCA overproduction in the Δ*bfd* cells are important and will be discussed below. 

### 2.2. Inhibition of Iron Mobilization from BfrB Affects Central Carbon Metabolism and Amino Acid Biosynthesis

**Figure 8:**
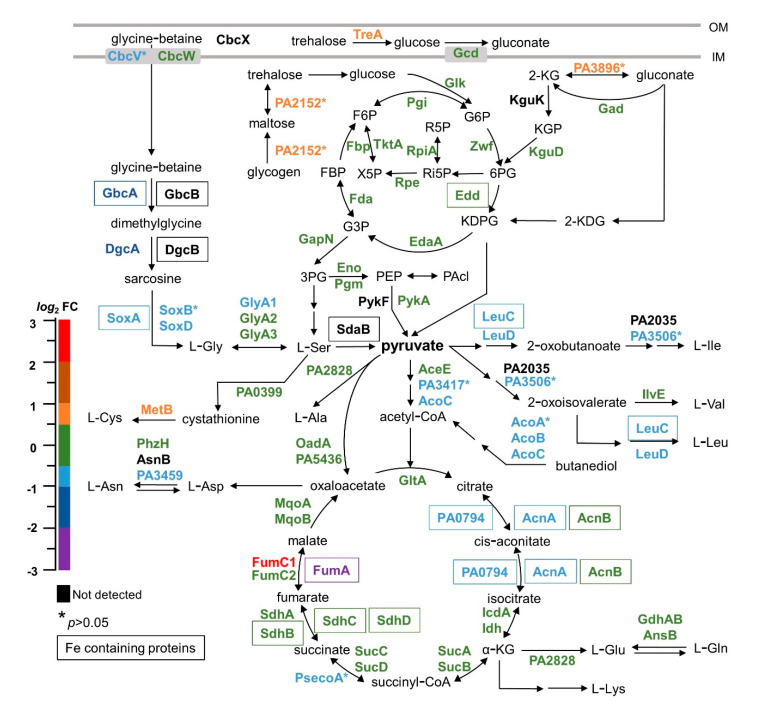
Proteins for carbon metabolism and for the biosynthesis of amino acids, whose skeletons are derived from the carbon and citrate cycles, are affected in the Δ*bfd* cells. The schematic represents the main pathways of carbon metabolism and some abbreviated pathways of amino acid biosynthesis. The relative abundances (Δ*bfd*/wt) of each of the proteins included in the scheme are color coded according to the *log*_2_FC scale. Enzymes that require iron to function are encased. The preparation of this scheme was aided by information from the bibliography, especially ref. [78,79]. 2-Ketogluconate (2-KG), 2-ketogluconate-6-phosphate (KGP), 2-keto-3-deoxy-D-glutamate (2-KDG), 6- phosphogluconate (6PG), ribulose-5-phosphate (Ri5P), ribose-5-phospahte (R5P), 2-keto-3-deoxy-6- phosphogluconate (KDPG), glyceraldehyde-3-phosphate (G3P), fructose-1,6-phosphate (FBP), fructose-6-phosphate (F6P).

Comparing the proteomic profiles of wt and Δ*bfd* cells shows significant differences in central carbon metabolism, which extend to the synthesis of several amino acids (Figure 8). We begin this discussion by pointing to the enrichment of enzymes involved in the conversion of glycogen to glucose (PA2152 and TreA) [72,73,74] in the Δ*bfd* cells. Glycogen is a major intracellular reserve polymer of the α-1-4-linked glucose monomers that accumulates in bacterial cells under conditions of limited growth, and it is degraded when carbon is limited [75,76,77,78,79]. As such, the enrichment of PA2152 and TreA in the Δ*bfd* cells suggests alterations in carbon metabolism. Previous studies comparing the metabolic flux of exponential phase *Pseudomonas putida* cells grown in iron replete vs. iron limiting conditions showed a decrease in metabolic flux through the Entner–Doudoroff route, as well as through Fe-containing metabolic enzymes of the tricarboxylic acid (TCA) cycle [80]. In agreement, our results show that several enzymes of the cycle are depleted in the Δ*bfd* cells (Figure 8). Enzymes in the upper portion of the TCA cycle which catalyze the conversion of pyruvate (AcoC, PA3417) or butanediol (AcoA, AcoB, AcoC) to acetyl-CoA [81] are depleted in the Δ*bfd* cells. In addition, the aconitase enzymes AcnA and PA0794, which depend on [4Fe-4S] catalytic centers to transform citrate to isocitrate via cis-aconitate, are also depleted in the mutant cells. In the bottom section of the TCA cycle, the conversion of fumarate to malate is probably affected by the depletion of FumA (an iron-dependent enzyme). The depletion of FumA in the mutant cells appears to be compensated by the significant enrichment of FumC1, an iron-independent fumarase hydratase, whose expression is known to be stimulated under iron limiting conditions [82].

The iron starvation caused by blocking the BfrB–Bfd complex also affects amino acid biosynthesis. Glycine plays an essential role as a precursor of proteins, nucleic acids and other metabolites. Glycine can be produced from choline via glycine-betaine (GB) and sarcosine, or from serine, as schematically depicted in Figure 8. *P. aeruginosa* can use GB as a sole source of carbon, nitrogen, and energy through a series of successive demethylation reactions that produce Gly [83,84]. Although the reasons are not yet clear, *P. aeruginosa* maintains intracellular pools of GB, the sizes of which are affected by the main carbon source, temperature or salinity [85]. Our results indicate that the GB pool is affected by iron limitation because both proteins required to convert GB to dimethylglycine (GbcA and GbcB) are iron-dependent; GbcA is significantly depleted in the Δ*bfd* cells and GbcB was not detected. The demethylation of dimethylglycine is catalyzed by DgcA and DgcB, and the product of the reaction, sarcosine, is known to stimulate the sarcosine oxidation and utilization regulator (SouR), thereby inducing the *sox*BDAG operon, along with a serine hydroxymethyl transferase (GlyA1) [86]. The SoxBDA enzymes catalyze the conversion of sarcosine to glycine, while GlyA1 catalyzes the catabolism of glycine to serine. Consistent with the depletion of DgcA and probable relatively low conversion of dimethylglycine to sarcosine in the mutant cells, the SoxBDA and GlyA1 enzymes are also depleted (Figure 8), suggesting that the assimilation of GB and sarcosine to Gly and Ser is adversely affected by the blockade of the BfrB–Bfd complex. As such, iron limitation in the Δ*bfd* cells adversely affects the pathways that transform GB and sarcosine into metabolites used for energy production and biosynthesis. It is probable that the sensitivity of this path to iron depletion starts with a fraction of the iron-dependent enzymes unable to assemble functional catalytic centers, initially affecting sarcosine biosynthesis, which in turn represses transcription of the SoxBDA and GlyA1 enzymes and affects the biosynthesis of Gly and Ser. 

**Figure 9:**
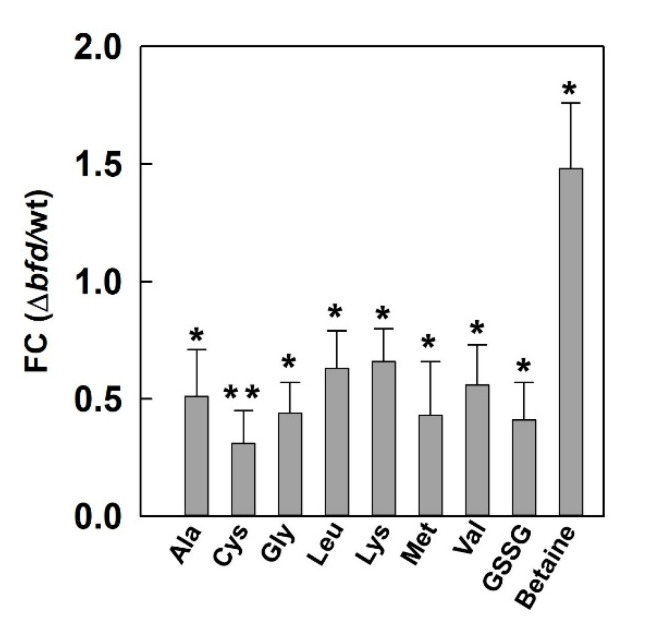
Amino acids derived from intermediates of the tricarboxylic acid (TCA) cycle are depleted in the Δ*bfd* cells. NMR spectroscopy-determined intracellular levels of amino acids and other metabolites in wt and Δ*bfd* cells are plotted as the fold change (FC) ratio Δ*bfd*/wt. Cystine and glutathione were measured as the oxidized forms, cystine and GSSG, respectively. *p* < 0.05 denoted by * and *p* < 0.01 by **.

The iron starvation elicited by blocking the BfrB–Bfd complex appears to also affect the biosynthesis of several other amino acids whose structures are formed from intermediates in the TCA cycle. Ala, Leu, Ile and Val are derived from pyruvate. The biosynthesis of Leu and Ile requires the large and small subunits of the iron-dependent 3-isopropylmalate dehydratase (LeuC and LeuD) and PA3506, which are depleted in the Δ*bfd* cells (Figure 8). The synthesis of Glu, Gln and Lys may also be impacted by depletion of the iron-dependent aconitase enzymes AcnA and PA0794, required to transform citrate to isocitrate. We used NMR spectroscopy (see Materials and Methods) to identify and measure the relative concentrations of several of these amino acids in the wt and Δ*bfd* cells. The results from these experiments (Figure 9, Table S3) demonstrate that the intracellular levels of Gly, Ala, Val, Leu, and Lys are depleted in the Δ*bfd* cells. Consequently, the observations made by comparing the proteomic profiles and the intracellular abundances of amino acids in the wt and Δ*bfd* cells provide strong support for the idea that iron limitation resulting from blocking the BfrB–Bfd complex impacts carbon metabolism and the biosynthesis of amino acids derived from metabolites in the carbon and citrate cycles. 

### 2.3. Inhibition of Iron Mobilization from BfrB Affects Proteins Involved in Respiration

**Figure 10:**
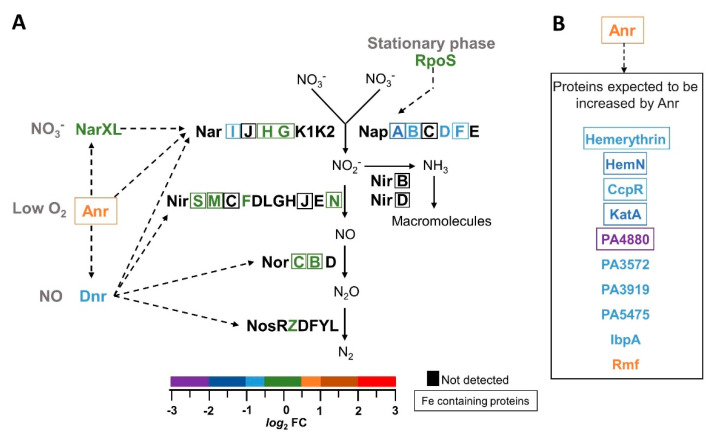
(**A**) Schematic representation of the proteins involved in the denitrification pathway. The relative abundances (Δ*bfd*/wt) of each of the proteins in the pathway are color coded according to the *log*_2_FC scale; proteins that require iron to function are encased. The conditions that activate expression of the regulatory proteins Anr, Dnr, NarXL and RpoS are in gray; the segmented arrows indicate which proteins are transcribed in response to each of the regulatory proteins. The preparation of this scheme was aided significantly by ref. [100]. (**B**) Proteins coded by genes expected to be transcriptionally activated by Anr are depleted in the Δ*bfd* cells, despite enrichment of Anr.

The blockade of the BfrB–Bfd complex also affected the abundances of several proteins involved in respiration (Figure 10), including the Anr (anaerobic regulation of arginine deaminase and nitrate reduction) and Dnr (dissimilatory nitrate respiration) regulators, and proteins assembling the membrane-bound and periplasmic nitrate reductases Nar and Nap. *P. aeruginosa* is well adapted for microaerobic respiration due to its branched aerobic respiratory chain terminated by five terminal oxidases; three are cytochrome c oxidases (cbb3-1, cbb3-2 and aa3), and two are cyanide-insensitive quinol oxidases (Cyo and CIO) [87]. *P. aeruginosa* cells, which are thought to prefer microoxic conditions, have been shown to grow at concentrations of dissolved oxygen less than 3 µM [88]. In fact, aerobically cultured *P. aeruginosa* cells block the transfer of O_2_ from the gas to the liquid phase, inducing microoxic conditions for cells in exponential and stationary phase cultures [89]. Under these conditions the regulator Anr stimulates transcription of cbb3-2, and is also responsible for the start of denitrification via the regulator Dnr, although Dnr-dependent pathways are not essential at this stage [90]. In the absence of O_2_, *P. aeruginosa* can use nitrate and nitrogen oxides as terminal electron acceptors. For this purpose, *P. aeruginosa* is equipped with three types of nitrate reductases: Nar, a membrane-bound enzyme expressed during anaerobic growth with nitrate as the terminal electron acceptor, Nap, a periplasmic enzyme expressed during the stationary growth phase in aerobic cultures, and Nas, a cytoplasmic enzyme which allows *P. aeruginosa* to use nitrate as a nitrogen source [87]. During anaerobic growth using nitrate for respiration, Nar and the enzymes Nir, Nor and Nos carry out the reduction of nitrate to N_2_. The regulators controlling these processes include Anr, which responds to low O_2_ tension, Dnr, which responds to Anr and nitric oxide (NO), and NarXL, which responds to Anr and nitrate (Figure 10A). Anr, Dnr and NarXL stimulate the transcription of the *nar*K1K2GHJI operon encoding nitrate/nitrite transporters (NarK1 and NarK2) and the structural genes of Nar [91]. The *nap* operon is thought to be under the control of RpoS, and therefore it is expressed in the stationary growth phase [87]. 

The Anr regulatory protein is enriched in the Δ*bfd* cells, whereas Dnr is depleted and NarXL exhibits no change relative to the wild type cells (Figure 10). A possible interpretation of these observations would suggest that the enrichment of Anr in the Δ*bfd* cells is in response to sensing lower O_2_ tension than in their wt type counterparts. The depletion of Dnr and NarI proteins, as well as the “normal” levels of NarXL, however, suggest that an alternative mechanism may be responsible for the Anr enrichment in Δ*bfd*, whereby the iron limitation imposed by blocking the BfrB–Bfd complex causes the accumulation of inactive Anr. In this context, it is important to note that Anr is an analog of *E. coli* FNR (fumarate nitrate reductase regulator) [92]. To function as a transcriptional regulator, FNR and Anr must be in dimeric form and harboring one O_2_-sensitive [4Fe-4S] cluster per subunit. Exposure to O_2_ causes iron loss from the [4Fe-4S] cluster, resulting in a cluster-free enzyme that dissociates into monomers unable to bind DNA [93]. Importantly, *E. coli* cells grown aerobically have been shown to contain significant amounts of apo-FNR [94], and the apo-FNR in these cells has been shown to be relatively stable and actively recycled into a functional [4Fe-4S] dimer under iron-replete conditions [95]. As such, the enrichment of Anr in the Δ*bfd* cells may be explained by the low iron and low sulfur (see below) conditions that slow the incorporation of iron and sulfur into [4Fe-4S] clusters in the existing and de novo synthesized Anr, causing the enzyme to accumulate in its inactive apo-form. Evidence supporting this idea was obtained from analysis of the proteomic profile in the context of Anr as a transcriptional activator of many genes in *P. aeruginosa* [96]. This analysis reveals that most of the proteins coded by genes transcriptionally activated by Anr are depleted in the Δ*bfd* cells, despite the enrichment of Anr (Figure 10B). Consequently, these observations support the hypothesis that iron limitation caused by blocking the BfrB–Bfd complex impairs the efficient incorporation of iron sulfur clusters into recycled or de novo synthesized apo-Anr. 

It is also of interest to note that among the proteins coded by genes transcriptionally activated by Anr but which are depleted in the Δ*bfd* cells, several are iron-dependent. Hemerythrin is a di-Fe center protein thought to transport O_2_ in microoxic conditions [97], HemN is a [4Fe-4S] enzyme that functions as an O_2_-independent coproporphyrinogen IX oxidase in heme biosynthesis, cytochrome *c* peroxidase (CcpR) is a di-heme enzyme thought to function in the prevention of peroxide buildup in the cell [98], and the cytoplasmic catalase KatA is a heme-containing enzyme that catalyzes peroxide decomposition into water and O_2_. The transcription of the *katA* gene is driven by two promoters; *katAp1* is required in the logarithmic growth phase, and *katAp2*, which is transcriptionally activated by Anr, is required for KatA function in the stationary phase [99]. These observations suggest that the biosynthesis and maturation of these important proteins is affected by low iron and sulfur, which limits the synthesis of iron- and sulfur-containing cofactors, and by the accumulation of inactive Anr, which leads to the repression of the corresponding genes.

Several of the proteins encoded by the *nap*EFDABC operon are also depleted in the Δ*bfd* cells (Figure 10A). The *nap* operon codes for the periplasmic nitrate reductase (Nap) enzyme and its accessory proteins. In aerobically grown *P. aeruginosa*, Nap expression is controlled by the sigma factor RpoS during the stationary growth phase [87]. Recent work has shown that nap expression is repressed in response to phenazines via a yet-unidentified regulator, with PCA and 5-Me-PCA exerting the stronger influence [100]. The same study reported that Anr is less active in the presence of phenazines, and suggested that phenazines can alter the balance between active and inactive Anr by oxidizing the [4Fe-4S] clusters of the active enzyme. These ideas are in good agreement with the observations reported herein, whereby iron starvation induced by blocking the BfrB–Bfd complex in the Δ*bfd* cells causes the enrichment of enzymes for the synthesis of phenazines (Figure 7A,B), as well as demonstrating high levels of PCA (Figure 7C). When this information is considered together, a model emerges wherein the elevated phenazine levels resulting from iron limitation (i) repress the *nap* operon and (ii) elevate the reduction potential of the cell, thus accelerating the oxidation of [4Fe-4S] centers, such as that in Anr. Iron limitation interferes with the efficient rebuilding of damaged [4Fe-4S] clusters, which results in the accumulation of inactive Anr and the concomitant repression of genes that are normally transcriptionally activated by functional Anr. 

These findings also suggest that it is probable that other enzymes whose functions depend on relatively sensitive [Fe-S] active centers can be affected similarly because the repair of the clusters is slowed or impeded by iron and sulfur limitation. In support of these ideas, below we present evidence suggesting that the blockade of the BfrB–Bfd complex also elicits a sulfur starvation response, which is expected to adversely affect the construction and repair of the [Fe-S] centers important for many crucial processes. 

### 2.4. Inhibition of Iron Mobilization from BfrB Affects Assimilatory Sulfate Reduction and Sulfur Traffic

**Figure 11:**
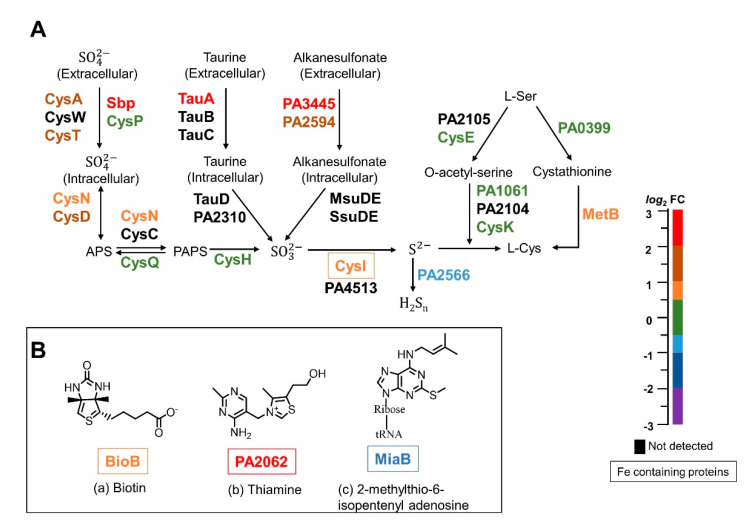
The sulfate starvation response proteins are enriched in the Δ*bfd* cells. (**A**) Schematic representation of the sulfate starvation-induced proteins. The relative abundances (Δ*bfd*/wt) of each of the proteins in the pathway is color coded according to the *log*_2_ FC scale; proteins that require iron to function are encased. (**B**) Proteins for the biosynthesis of sulfur-containing cofactors are affected in the Δ*bfd* cells.

Sulfur, an essential element for bacterial life, is a component of the amino acids cysteine and methionine. *P. aeruginosa* assimilates sulfur preferentially from sulfate (${SO}_{4}^{2-}$), which is imported from the environment and reduced intracellularly to sulfide ($S^{2-}$) for its subsequent incorporation into sulfur-containing molecules. A primary path of sulfur assimilation, the incorporation of $S^{2-}$ into cysteine plays a crucial role in the biogenesis of [Fe-S] clusters, in the biosynthesis of antioxidant proteins and peptides, and as a precursor of methionine, coenzyme A, coenzyme M, and also thiamine, biotin, lipoic acid and the siderophore pyochelin [101,102]. Bacteria respond to sulfate or cysteine starvation by deploying the so-called sulfate starvation-induced proteins (SSI proteins), which in *P. aeruginosa* consist of sulfate and sulfonate transport systems for the uptake of sulfate and alkyl sulfonates, as well as sulfatase and sulfonatase enzymes, which function in the reduction of the sulfate and alkylsulfonate to $S^{2-}$ for its subsequent incorporation in cysteine [103]. In this context, it is interesting that many of the SSI proteins are enriched in the Δ*bfd* cells (Figure 11A), because it indicates that the mutant cells are deficient in sulfate or cysteine. The SSI proteins are coded by genes in the *spb*-containing *cys*PTWA operon, where Sbp is a periplasmic sulfate-binding protein, CysP is a periplasmic thiosulfate binding protein, CysT and CysW are the membrane proteins, and CysA is an ATPase. That Sbp is highly enriched whereas CysP is not suggests that the Δ*bfd* cells are actively searching/importing ${SO}_{4}^{2-}$ [104]. The cytoplasmatic enzymes CysD and CysN, also enriched in the mutant cells, catalyze the transformation of ${SO}_{4}^{2-}$, first into adenylyl sulfate (APS) and then into 3′-phosphoadenylyl sulfate (PAPS). PAPS is then transformed into sulfite (${SO}_{3}^{-}$) by the enzyme CysH, and ${SO}_{3}^{-}$ is subsequently reduced to $S^{2-}$ by the NADPH-dependent sulfite reductase CysI, which requires siroheme and FAD to function [105]. Equally consistent with a sulfur starvation response, the proteomic data indicate the enrichment of TauA (taurine uptake) and enzymes PA3445 and PA2594, which function in the uptake of alkanesulfonates [105] (Figure 11A).

The enrichment of proteins in the sulfate starvation response in the Δ*bfd* cells indicates that the iron limitation imposed by blocking the BfrB–Bfd complex leads to ${SO}_{4}^{2-}$ deficiency. Given that sulfur assimilation proceeds via the reduction of ${SO}_{4}^{2-}$ for the subsequent incorporation of a sulfur atom from $S^{2-}$ in cysteine (and other organic molecules), the enrichment pattern suggests cysteine deficiency in the Δ*bfd* cells. In this context, it is interesting that $S^{2-}$(in equilibrium with ${\mathrm{HS}}^{-}$ and $H_{2}S$ ) can be oxidized to sulfane sulfur (HS_n_H; *n* ≥ 1) by a sulfide:quinone oxidoreductase enzyme [106,107]. The pertinent sulfide:quinone oxidoreductase enzyme in *P. aeruginosa* (PA2556) is depleted in the Δ*bfd* cells (Figure 11A), an observation indicating that the Δ*bfd* cells prioritize $S^{2-}$ for cysteine biosynthesis. Additional evidence supporting the idea of cysteine deficiency was obtained from the NMR spectroscopic analysis, which shows that the levels of cysteine (measured as cystine) and glutathione (measured as oxidized form) in the Δ*bfd* cells are ~30–40% of those in the wt cells (Figure 9). It is therefore clear that the iron starvation induced by blocking the BfrB–Bfd complex leads to cysteine deficiency in *P. aeruginosa*. Cysteine deficiency in the iron-starved Δ*bfd* cells is probably a consequence of several processes, including but not restricted to: (i) the overproduction of pyochelin, which requires two cysteine molecules in its biosynthesis, (ii) the stress caused by an abundance of phenazines, such as the high-redox potential PCA, which induces oxidative stress and therefore the faster turnover and damage of sulfur-dependent oxidative stress defense molecules, such as glutathione and thioredoxin, and (iii) the repair and de novo synthesis of [Fe-S] clusters oxidized by the abundance of high-redox potential phenazines. 

Additional evidence indicating that iron starvation in the Δ*bfd* cells affects sulfur management stems from observations regarding the biosynthesis of cofactors containing sulfur atoms, such as biotin, thiamine, and tRNA thiolation (Figure 11B). In the final step of biotin biosynthesis, the incorporation of a sulfur atom requires biotin synthase (BioB), which is enriched in the Δ*bfd* cells. The sulfur atom incorporated into biotin is donated from a [2Fe-2S] cluster in BioB, which leads to cluster loss and inactive enzyme, followed by an iron- and sulfur-dependent repair mechanism that installs a new [2Fe-2S] cluster in BioB [108]. As such, it is possible that the iron and sulfur deficiencies in the Δ*bfd* cells lead to an enrichment of BioB as a mixture of active and inactive forms. The enzyme PA2062, a cysteine desulfurase akin to IscS, is implicated in thiamine metabolism and is highly enriched in the Δ*bfd* cells (Figure 11B). Some organisms have more than one copy of cysteine desulfurase, which play pivotal roles in the initial stages of sulfur trafficking within cells. These enzymes extract a sulfur atom from cysteine to form a persulfide intermediate, which is subsequently incorporated into the biosynthetic pathways leading to the formation of [Fe-S] clusters and sulfur-containing biofactors [109]. The enzyme MiaB, depleted in the Δ*bfd* cells, catalyzes the posttranscriptional thiolation of a subset of tRNAs, a process required for their maturation. MiaB harbors two essential [4Fe-4S] clusters, which catalyze the insertion of a sulfur atom into isopentenyl adenosine to produce 2-methylthio-6-isopentenyl adenosine [110] (Figure 11B). 

## 3. Conclusions

Our work aims at introducing a new strategy for exploiting bacterial iron metabolism, the inhibition of iron mobilization from bacterioferritin. Iron mobilization from BfrB in *P. aeruginosa* requires the formation of a specific complex with Bfd (Figure 1) [27,30]. Accordingly, blocking iron mobilization from BfrB by deletion of the *bfd* gene (Δ*bfd*) “locks” iron in the bacterioferritin [16] and impairs biofilm formation [14]. These findings encouraged the development of novel small molecule inhibitors of the BfrB–Bfd complex, which constrain *P. aeruginosa* cell growth and potentiate the activity of the fluoroquinolone antibiotics [10]. Culturing Δ*bfd* cells, or wt cells in the presence of an inhibitor of the BfrB–Bfd complex, leads to pyoverdine overproduction, which has been interpreted to suggest that “locking” iron in BfrB causes intracellular iron limitation [10,16]. Although pyoverdine overproduction indeed suggests iron limitation, secretion of the siderophore may occur independently of its regulation by intracellular iron levels [20], and does not per se indicate the extent of intracellular iron limitation, or if such limitation affects cellular fitness. 

We conducted this work to investigate if the deletion of the *bfd* gene elicits iron homeostasis dysregulation and if the lesion in iron metabolism (Δ*bfd*) affects metabolic homeostasis in *P. aeruginosa*. The results unequivocally show that the Δ*bfd* cells experience severe iron limitation, as is evident not only in the overexpression of proteins involved in siderophore biosynthesis, but also in the enrichment of proteins involved in heme uptake/utilization, and AQ and phenazine biosynthesis. The analytical quantification of pyoverdine, AQs and phenazines corroborated our interpretation of the proteomic response and demonstrated that all these metabolites are produced at significantly higher levels in the Δ*bfd* cells. In the context of the intracellular iron starvation that ensues in the Δ*bfd* cells, it is important to underscore that the cells were cultured in iron-replete media (10 µM Fe), so the acute iron starvation that develops in the Δ*bfd* cells is a direct consequence of iron flowing unidirectionally into BfrB. 

In addition to demonstrating global iron limitation, the proteomic profiles show that the lesion in iron metabolism caused by locking iron in BfrB adversely affects important metabolic pathways, including carbon metabolism, amino acid biosynthesis, respiration, and sulfur metabolism. Iron limitation caused by inhibiting iron mobilization from BfrB appears to affect these paths in at least two predominant ways, as follows: (i) It affects the abundance of iron-dependent proteins, which in turn affect the regulation and abundance of other proteins participating in the same metabolic pathways. (ii) It elicits an overproduction of high-potential phenazines, which induce oxidative stress and consequently damage to [Fe-S] clusters and sulfur-dependent oxidative stress defense molecules, such as glutathione and thioredoxin. The increased demand for sulfur needed to repair [Fe-S] clusters and sulfur-dependent molecules probably overwhelms the sulfur homeostasis machinery, as is suggested by the depleted levels of cysteine and glutathione in the Δ*bfd* cells.

Finally, it is interesting to compare the findings reported herein with results from a study by Nelson et al. comparing the proteomic responses of wt *P. aeruginosa* PAO1 cells cultured in Fe-rich and Fe-deficient media [111]. It is important to underscore that in the study by Nelson et al., the cellular response to low iron was investigated by comparing the proteomic response of wt cells with intact iron homeostasis machinery cultured in high vs. low iron conditions. In comparison, in this study we compare the proteomic response of wt cells with that of Δ*bfd* cells, which have a lesion in the iron homeostasis machinery. In addition, in this study the cells were cultured under iron-replete conditions; intracellular iron limitation in the Δ*bfd* cells develops because intracellular iron is locked in BfrB. A comparison of the proteins detected in each of the studies and their corresponding abundances is presented in Table S2. Although in both studies iron limitation elicits the deployment of siderophores and heme acquisition/utilization systems, considering the differences observed in the two studies is insightful. In the wt cells cultured under low iron conditions (Nelson et al.), the proteins involved in sulfate and sulfonate assimilation, the reduction of the oxo-anions to sulfite and sulfide, and the biosynthesis of cysteine, are depleted [111]. In stark contrast, these proteins are enriched in the Δ*bfd* cells (Figure 11). A second significant difference is in phenazine biosynthesis. While in the wt cells cultured in low iron conditions the proteins involved in phenazine biosynthesis are depleted [111], these proteins are enriched in the Δ*bfd* cells (Figure 7). Considering these two significant differences supports the detrimental consequences of dysregulating iron homeostasis by blocking the BfrB–Bfd complex; the overproduction of phenazines in the Δ*bfd* cells leads to oxidative stress, which in turn damages [Fe-S] clusters and sulfur-containing antioxidant molecules. This situation imposes a very difficult-to meet-demand for sulfur and iron assimilation, which ultimately results in low levels of cysteine, antioxidant molecules and sulfur- and iron-dependent molecules (Figure 9 and Table S1). These observations lead us to hypothesize that small molecule inhibitors of the BfrB–Bfd complex, by virtue of eliciting an irreversible accumulation of iron in BfrB, would trigger a homeostatic dysregulation akin to that reported here for the Δ*bfd* cells, thus encouraging the development of inhibitors of the BfrB–Bfd complex for possible therapeutic applications.

## 4. Materials and Methods

### 4.1. Strains, Media and Growth Conditions

Chemicals were purchased from Fisher Scientific (Waltham, MA, USA) unless otherwise stated. *P. aeruginosa* (PA01) was purchased from the University of Washington Genome center (Seattle, WA, USA). The PA01-derived strain with an unmarked, in-frame deletion of the *bfd* gene (*Δbfd)* had been prepared previously [16]. All strains were maintained on Pseudomonas Isolation Agar (PIA, BD Biosciences, San Jose, CA, USA). *Pseudomonas aeruginosa* isolation (PI) media was used to culture cells. PI media contains 20 g L^−1^ peptone, 1.4 g L^−1^ MgCl_2_·6H_2_O, 10 g L^−1^ K_2_SO_4_, 25 mg L^−1^ irgasan (Sigma-Aldrich, St. Louis, MO, USA), and 20 mL L^−1^ glycerol, pH 7.0. PI media was supplemented with 10 µM iron using a 10 mM stock of (NH_4_)_2_Fe(SO_4_)_2_ (pH~2.0).

### 4.2. Cell Growth and Preparation of Lysate Solution

Pre-cultures (5 mL) of wild type (wt) and Δ*bfd P. aeruginosa* cells were grown in PI media for 12 h in 50 mL conical tubes covered with an air-permeable membrane at 37 °C and 220 rpm. The cells were centrifuged at 4000 rpm for 10 min and the cell pellets washed twice with PI media not supplemented with iron. The cell pellets were resuspended in PI media supplemented with 10 µM iron, diluted in the same media to an optical density at 600 nm (OD_600_) = 0.001, and 50 mL of the cell suspension was transferred to 250 mL polypropylene Erlenmeyer flasks. The flasks were covered with an air permeable membrane and incubated for 30 h at 37 °C and 230 rpm. A small volume was sampled for enumeration of viable cells prior to harvesting the cells by centrifugation (4000 rpm, 15 min and 4 °C). The cell pellets were washed twice with 10 mL of phosphate buffer saline (PBS) and stored at −20 °C overnight. The cell pellets were re-suspended in 5 mL 1% SDS in water and sonicated for 90 s in a Qsonica Q500 sonicator operating at a 20% pulse amplitude, alternating 10 s pulse-on and 10 s pulse-off. The resultant lysate solutions were stored at −20 °C until further analysis. The cell-free supernatants were analyzed for pyoverdine using a previously reported protocol [16]. 

### 4.3. Sample Preparation and Proteomics Workflow

These were carried out by following the published protocols developed at the Proteomics Core Facility, Louisiana State University Health Science Center, New Orleans [112]. Three biological replicates were conducted each for the wt and Δ*bfd* cells. Briefly, 100 µg of protein per sample was used for trypsin digestion. The protein concentration was determined with the aid of BCA protein assay kit (Pierce, Thermo Scientific, Rockford, IL, USA) using an eight-point BSA standard curve. Proteins were reduced using 10 mM tris(carboxyethyl)phosphine (TCEP) for 1 h at 55 °C, and subsequently alkylated with 20 mM iodoacetamide for 30 min at room temperature in the dark. Proteins were subjected to chloroform-methanol precipitation, and the resultant pellet was digested with 2 µg of sequencing grade trypsin at 37 °C overnight. The next day, tryptic peptides were labeled with a TMT 6plex reagents set (Thermo Scientific) according to the manufacturer’s recommended protocol. The tagged samples were stored at −80 °C until analysis. An equal volume from each TMT tagged sample was pooled into a single tube and purified using acidic reverse phase conditions (SepPak-Waters, Milford, MA, USA). Once the samples were dried, a fractionation step was carried out to lower the complexity of the sample. The sample volume was adjusted to 115 µL by 20 mM ammonium hydroxide (pH 10), and basic-pH reverse phase chromatography (Dionex U3000, Thermo Scientific, West Palm Beach, FL, USA) was carried out according to the parameters below. The injection volume was 100 µL, and the flow rate was 0.1 mL/min. A 90 min gradient was developed from 10 mM ammonium hydroxide (pH = 10) to 100% acetonitrile (pH = 10). A total of 48 200 µL fractions were collected, and the fractions were re-combined in a checkerboard fashion (fraction 1, 13, 25, and 37—super fraction 1; fractions 2, 14, 26, 38—super fraction 2, etc.), resulting in 12 ‘super fractions’. These fractions were run on a Dionex U3000 nanoflow coupled to a Thermo Scientific™ Orbitrap Fusion™ Tribrid™ Mass Spectrometer (Thermo Scientific). Liquid chromatography was performed at a flow rate of 0.3 µL/min (Trap column: C18 PepMap 100, 5 µm, 100 Å; separation column: PicoChip REPROSIL-Pur C18-AQ, 3 µm, 120 Å, 105 mm). A 90 min chromatographic method was employed. The gradient was as follows: 2–25% ACN in 0.1% formic acid (FA)-65 min, 50% ACN/FA-10 min, 90% ACN/FA-5 min, and re-equilibration 2% ACN/FA-10 min. 

Electrospray ionization was performed at 2.6 kV. Data acquisition used an MS3 approach; survey scans were performed in the Orbitrap, using a resolution of 120,000. MS2 scans were done in the linear ion trap, using a collision-induced dissociation of 25%. For the fragmentation of the TMT-reporter ions, high-energy collision dissociation (HCD) of 65% was used and detected in the Orbitrap using a 30,000 resolution. Runs were done in triplicate for each super fraction.

TMT data were analyzed using Proteome Discoverer 2.2. The triplicate runs for the 12 super fractions were merged and searched against SEQUEST. The parameters for searching included static modifications of TMT reagents on lysine and N-terminus (+229.163), carbamidomethyl on cysteines (=57.021), and the dynamic modification of methionine oxidation (=15.9949), two maximum trypsin missed-cleavages, parent ion tolerance—10 ppm, and the fragment mass tolerance—0.6 Da. High-scoring peptides were considered using a false discovery rate of 1%. Data files were searched against the *Pseudomonas* Genome Database (www.Pseudomonas.com). 

### 4.4. Data Analysis

A volcano plot was generated using the *log*_2_ (fold change) and the -*log*_10_ (abundance ratio *p*-values). Data were filtered based on the fold change, and the abundance ratio *p*-values. Proteins exhibiting a (Δ*bfd*)/(wild type) fold change ≥1.4 and ≤0.7, and the abundance ratio *p*-value < 0.05, are considered to be differentially expressed [34]. The filtered datasets were uploaded onto the Kyoto Encyclopedia of Genes and Genomes (KEGG) Pathway Mapper and searched against *P. aeruginosa* (PA01) organism-specific pathways [113]. Identified proteins in the KEGG mapper were also searched against the *Pseudomonas* genome database (https://www.pseudomonas.com) and UniProt (https://www.uniprot.org) to obtain functional predictions and classifications [36].

### 4.5. NMR Analysis of Metabolites

*Pseudomonas aeruginosa* PAO1 cells (three biological replicates) were cultured and harvested as described above. The harvested cells were resuspended in 1.25 mL of HPLC-grade methanol (OmniSolv), incubated for 30 min at ambient temperature and then centrifuged for 30 min at 4000 rpm and 4 °C. The supernatants were transferred to separation funnels containing 1.25 mL of deionized water and then extracted with 2.5 mL of chloroform. The aqueous phase was frozen at −80 °C and freeze-dried with the aid of a SpeedVac concentrator (Thermo SAVANT, SPD111V) overnight. The dry powder was dissolved in 550 µL of PBS (pH 7.4) prepared in D_2_O (99.8%) containing 0.01% (*w/v*) 3-(Trimethylsilyl) propane-1-sulfonate, sodium salt (DSS) for chemical shift referencing. The solutions were vortexed and centrifuged and then transferred to 5 mm NMR tubes. 

NMR spectra were acquired at 298 K on a Bruker Avance III 800 MHz spectrometer equipped with a 5 mm TCI cryoprobe. One-dimensional ^1^H NMR spectra were acquired (128 scans) using the 1D-NOESY experiment and pre-saturation of the water peak, with a spectral width of 16,000 Hz, 65 K data point, 2.0 s acquisition time and 1.0 s relaxation delay. Spectra were apodized with a 0.3 Hz line broadening prior to Fourier transformation. ^1^H-^13^C HSQC (heteronuclear single quantum correlation) spectra were acquired with a spectral width of 8000 Hz, 4096 data points and 1.0 s relaxation delay in the direct-detected dimension (128 scans), and 512 data points over a spectral width of 36,000 Hz in the indirect-detected dimension. ^1^H-^1^H TOCSY (total correlation spectroscopy) spectra were acquired with a spectral width of 8000 Hz, 4096 data points and 1.0 s relaxation delay (16 scans) in the direct-detected dimension, and 256 datapoints over a spectral with of 8000 Hz in the indirect-detected dimension. Adjustments to the baseline, peak phasing and chemical shift referencing were made using Bruker Topspin 3.6.2. To identify the identity of metabolites, the chemical shifts in the NMR spectra were compared to the Biological Magnetic Resonance Database [114] and the Human Metabolome Database [115] for positive matches. To corroborate metabolite identifications the chemical shifts were correlated using the ^1^H-^13^C HSQC and the ^1^H-^1^H TOCSY spectra (Table S3). Combining the information from the 1D and 2D NMR spectra greatly reduced the ambiguity of assigning chemical shifts to metabolites. The volumes of judiciously chosen peaks in the ^1^H-^13^C HSQC spectra were integrated and used to compare the relative levels of metabolites in the wt and Δ*bfd* cells. The average and standard deviations obtained from 3 biological replicates have been summarized in Figure 9. The statistical significance between the means and standard deviations of values comparing the levels of metabolites in the wt and Δ*bfd* cells was determined using Student’s *t* test with the aid of SigmaPlot (Systat Software, Inc. San Jose, CA, USA).

### 4.6. Analysis of 2—Alkyl-4-Quinolones (AQs) 

The HHQ, PQS and HQNO extracted from bacterial cells and cell culture supernatants were quantitated with the aid of liquid chromatography—mass spectrometry using a previously described method [14]. In brief: For the analysis of AQs in bacterial cells, a 1.0 mL sample of bacterial cell culture was centrifuged and the cell pellets resuspended in 3 mL HPLC-grade methanol. For the analysis in spent media, 3 mL of cell culture were clarified by centrifugation. The solutions were acidified with 15 µL 12 N HCl, extracted into 6 mL of ethyl acetate and incubated at ambient temperature for 1 h with slow shaking. The organic layers were separated by centrifugation, transferred to new micro centrifuge tubes, and evaporated overnight using a SpeedVac Concentrator. Quantitative analysis was performed using an LC-MS system comprising an Agilent 1260 Infinity II HPLC and an Agilent 6230 time-of-flight mass spectrometer operating in the positive ion mode with a capillary voltage of 4000 V, end plate voltage of 150 V, and nebulizing gas (N_2_) temperature of 325 °C. Samples were separated in a Poroshell 120 EC-C8 column (3.0 × 100 mm, 2.7 µm particle size, 120 Å pore size; Agilent) with a 0.4 mL/min flow rate and the following gradient: 97% A (2 min), 63% A (5 min), 57% A (21 min), and 3% A (31 min), revert to the initial conditions and equilibrate for 11 min; A: 0.1% formic acid in water. B: 0.1% formic acid in acetonitrile. AQs were detected using full scan mode (*m/z* 130 to 350) and quantitated by measuring the integrated peak area of the corresponding [M + H]^+^ ions and a standard curve constructed from commercially available HHQ, HQNO, and PQS. 

### 4.7. Analysis of Phenazine 1-Carboxylic Acid (PCA) and Pyocyanin (PYO)

The cell suspension (3 mL) was acidified to pH 4.0 with HCl prior to extraction with 6 mL of ethyl acetate. The ethyl acetate extracts were kept at room temperature for 30 min with gentle shaking prior to centrifugation at 4 °C and 4000 rpm for 10 min. The ethyl acetate layer was transferred to new microcentrifuge tubes and evaporated with the aid of a Speed Vac Concentrator. Dried samples were resuspended in 300 µL of methanol and stored at −20 °C. Quantitative determination was carried out by LC-MS using an Agilent 6230 TOF Ms (see above) in the positive ion mode. Samples were separated in a Poroshell 120 EC-C18 column (2.1 × 100 mm, 2.7 μm particle size, 120 Å pore size; Agilent) using a flow rate of 0.4 mL/min and the following gradient: 95% A (2 min), 40% A (11 min), 5% A (26 min), and reequilibration to initial conditions (11 min); A = 0.1% formic acid in water, B = 0.1% formic acid in acetonitrile. Phenazines were detected using full scan mode (*m/z* 130 to 350) and quantitated by measuring the integrated peak area of the corresponding [M + H]^+^ ions and a standard curve constructed using commercially available PCA (Evo Blocks Ltd., Budapest, Hungary) and PYO (Cayman Chemical, Ann Arbor, MI, USA).

## Supplementary Materials

The following are available online at https://www.mdpi.com/2076-0817/9/12/980/s1. Figure S1: Growth curves and levels of pyoverdine secreted by wt and Δ*bfd P. aeruginosa* cells; Table S1: Proteins exhibiting significant abundant differences between wt and Δ*bfd* cells; Table S2: Proteins exhibiting significant abundance differences between wt and Δ*bfd* cells compared to proteins exhibiting significant abundance differences between wt cells cultured in high vs. low iron conditions; Table S3: Resonance assignments for ^1^H and ^13^C chemical shifts of amino acids and other metabolites identified by NMR spectroscopy in the wt and Δ*bfd* cells.
