# Supplementary material for: Mobilization of Iron Stored in Bacterioferritin Is Required for Metabolic Homeostasis in Pseudomonas aeruginosa
Source: Pathogens. 2020 Nov 24;9(12):980. doi: 10.3390/pathogens9120980 (PMC7760384; doi:10.3390/pathogens9120980)
Supplement: Supplementary file 1 [file pathogens-09-00980-s001.pdf]

SUPPORTING INFORMATION

# Mobilization of Iron Stored in Bacterioferritin is Required for Metabolic Homeostasis in *Pseudomonas aeruginosa*

Achala N. D. Punchi Hewage [1], Leo Fontenot [2], Jessie Guidry [3], Thomas Weldeghiorghis [2], Anil K. Mehta [4], Fabrizio Donnarumma [2], and Mario Rivera [2,*]

[1] Department of Chemistry, University of Kansas, 2030 Becker Dr., Lawrence, KS, 66047, USA; achala@ku.edu

[2] Department of Chemistry, Louisiana State University, 232 Choppin Hall, Baton Rouge, LA, 70803, USA; lfont39@lsu.edu (L.F); thomaskw@lsu.edu (T.W); fabrizio@lsu.edu (F.D)

[3] Department of Biochemistry and Molecular Biology, Louisiana State University Health Science Center, 1901 Perdido Street, New Orleans, LA, 70112, USA; jjguid@lsuhsc.edu

[4] National High Magnetic Field Laboratory, University of Florida, 1149 Newell Drive, Gainesville, FL, 32610, USA; anil.mehta@ufl.edu.

*Corresponding author. E-mail: mrivera@lsu.edu

ORCID: 0000-0002-5692-5497

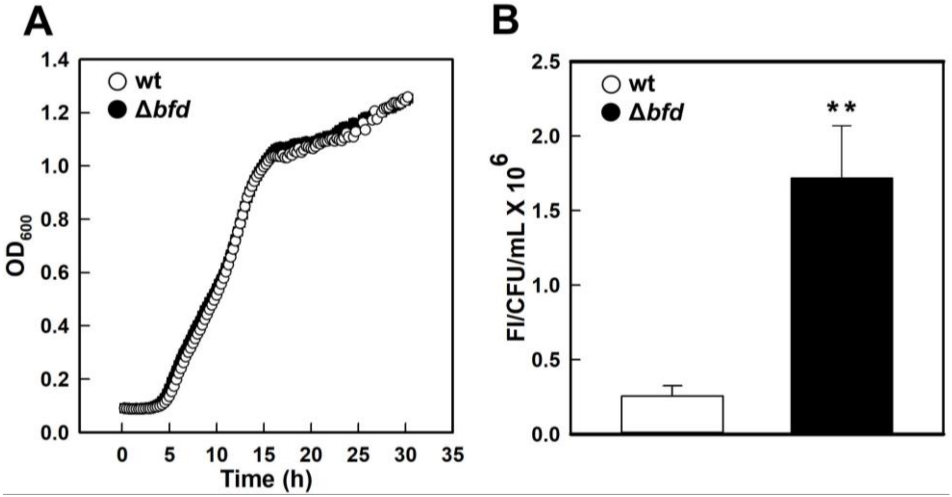


**Figure S1.** Growth curves and levels of pyoverdine secreted by wt and Δ*bfd* *P. aeruginosa* cells. (**A**) *P. aeruginosa* cells (wt and Δ*bfd*) were cultured in PI media supplemented with 10 μM Fe at 37 °C and shaking at 220 rpm. For the purpose of all the analyses reported in this work, the cells were harvested by centrifugation 30 h post inoculation. (**B**) Pyoverdine secreted by the cells was measured in the cell-free supernatants by acquiring fluorescence emission spectra (430-550 nm) with excitation at 400 nm (10 nm slit width) and emission at 460 nm (10 nm slit width). Fluorescence intensity normalized to viable cell count (CFU/mL) shows that the Δ*bfd* cells secrete approximately sixfold more pyoverdine than the wt cells. $p < 0.01$ denoted by ** relative to wt.

**Table S1**: Proteins exhibiting significant abundance differences between wt and Δ*bfd* cells.

| Protein ID | Name | Function | Metal binding | FC = Δ*bfd*/wt | *p* value |
|---|---|---|---|---|---|
| | | | | *log*$_2$FC | |
| **Pyoverdine biosynthesis** | | | | | |
| PA2385 | PvdQ | Acyl-homoserine lactone acylase | | 3.14 | 3.9E-10 |
| PA2386 | PvdA | L-ornithine N(5)-monooxygenase | | 3.02 | 1.2E-15 |
| PA2388 | FpvR | | | -2.18 | 1.7E-03 |
| PA2389 | PvdR | | | 2.81 | 2.8E-06 |
| PA2390 | PvdT | Macrolide export ATP-binding/permease protein | | 2.73 | 2.0E-10 |
| PA2391 | OpmQ | Probable outer membrane protein | | 2.00 | 1.5E-04 |
| PA2392 | PvdP | | | 4.11 | 5.2E-10 |
| PA2393 | PvdM | Probable dipeptidase | | 3.22 | 1.3E-13 |
| PA2394 | PvdN | | | 3.61 | 2.3E-11 |
| PA2395 | PvdO | | | 3.48 | 7.9E-16 |
| PA2396 | PvdF | Pyoverdine synthetase F | | 3.25 | 1.1E-10 |
| PA2397 | PvdE | Pyoverdine biosynthesis protein | | 3.16 | 1.6E-12 |
| PA2398 | FpvA | Ferripyoverdine receptor | | 1.72 | 3.3E-07 |
| PA2399 | PvdD | Pyoverdine synthetase D | | 2.75 | 1.8E-13 |
| PA2400 | PvdJ | Probable non-ribosomal peptide synthetase | | 2.87 | 1.4E-12 |
| PA2402 | PvdI | Pyoverdine peptide synthetase | | 2.87 | 4.6E-18 |
| PA2403 | FpvG | Uncharacterized protein | | 2.90 | 6.8E-12 |
| PA2404 | FpvH | Uncharacterized protein | | 2.52 | 1.8E-04 |
| PA2405 | FpvJ | Probable adhesion protein | | 2.94 | 1.6E-11 |
| PA2407 | FpvC | Probable ATP-binding component of ABC transporter | | 2.97 | 1.6E-09 |
| PA2408 | FpvD | Uncharacterized protein | | 1.20 | 1.1E-03 |
| PA2410 | FpvF | Probable thioesterase | | 2.44 | 1.9E-09 |
| PA2411 | | MbtH domain-containing protein | | 2.73 | 6.3E-10 |
| PA2412 | | L-2,4-diaminobutyrate:2-ketoglutarate 4-aminotransferase | | 1.20 | 4.5E-03 |
| PA2413 | PvdH | L-sorbosone dehydrogenase | | 3.10 | 6.2E-19 |
| PA2424 | PvdL | | | 2.76 | 6.2E-20 |
| PA2425 | PvdG | | | 1.75 | 6.3E-05 |
| PA2426 | PvdS | Sigma factor | | 0.91 | 4.8E-03 |
| PA4168 | FpvB | Second ferric pyoverdine receptor FpvB | | 0.67 | 2.9E-01 |
| **Pyochelin biosynthesis** | | | | | |
| PA4218 | FptX | Probable transporter | | 3.14 | 2.1E-08 |
| PA4219 | YfpB | Uncharacterized protein | | 2.83 | 9.1E-11 |
| PA4221 | FptA | Fe(3+)-pyochelin receptor (Fe(III)-pyochelin receptor) | | 2.50 | 2.9E-10 |
| PA4222 | PchI | Probable ATP-binding component of ABC transporter | | 3.36 | 8.1E-19 |
| PA4223 | PchH | Probable ATP-binding component of ABC transporter | | 3.32 | 1.8E-11 |
| PA4224 | PchG | Pyochelin biosynthetic protein | | 2.74 | 3.2E-21 |
| PA4225 | PchF | Pyochelin synthetase | | 2.38 | 9.0E-23 |
| PA4226 | PchE | Dihydroaeruginoic acid synthetase | | 2.79 | 9.9E-17 |
| PA4227 | PchR | Regulatory protein | | 1.19 | 4.1E-06 |

| | | | | | |
|---|---|---|---|---|---|
| PA4228 | PchD | Pyochelin biosynthesis protein | | 2.84 | 3.4E-17 |
| PA4229 | PchC | Pyochelin biosynthetic protein | | 1.14 | 1.0E-07 |
| PA4230 | PchB | Isochorismate pyruvate lyase | | 3.10 | 6.1E-18 |
| PA4231 | PchA | Salicylate biosynthesis isochorismate synthase | | 1.93 | 1.2E-05 |
| **Heme iron acquisition** | | | | | |
| PA0672 | HemO | Heme oxygenase | | 1.49 | 6.9E-13 |
| PA4708 | PhuT | Heme-transport protein | | 1.42 | 2.3E-04 |
| PA4709 | PhuS | | | 1.23 | 3.6E-04 |
| PA4710 | PhuR | Heme/Hemoglobin uptake outer membrane receptor | | 2.59 | 1.5E-14 |
| **Other iron acquisition and transport related proteins** | | | | | |
| PA0471 | FiuR | Probable transmembrane sensor | | 0.66 | 5.0E-02 |
| PA2466 | FoxA | Ferrioxamine receptor FoxA | | 0.56 | 7.5E-04 |
| PA3901 | FecA | Fe(III) dicitrate transport protein FecA | Fe | 0.81 | 1.2E-01 |
| PA4514 | PiuA | Probable outer membrane receptor for iron transport | | 1.02 | 1.6E-04 |
| PA4675 | OptH | Probable TonB-dependent receptor | | 0.53 | 4.8E-01 |
| **Quorum sensing** | | | | | |
| PA0996 | PqsA | Anthranilate--CoA ligase | | 1.71 | 1.4E-03 |
| PA0997 | PqsB | Hypothetical protein | | 2.06 | 1.6E-06 |
| PA0998 | PqsC | Hypothetical protein | | 2.28 | 1.1E-04 |
| PA0999 | PqsD | 3-oxoacyl-ACP synthase | | 2.39 | 7.2E-11 |
| PA1000 | PqsE | Thioesterase PqsE | | 2.08 | 9.6E-03 |
| PA1871 | LasA | Protease LasA (Staphylolytic protease) | | 0.55 | 8.2E-02 |
| PA2569 | | Uncharacterized protein | | 0.78 | 4.3E-02 |
| PA2570 | LecA | PA-I galactophilic lectin (PA-IL) (Galactose-binding lectin) | | 1.53 | 1.3E-02 |
| PA3476 | RhlI | Acyl-homoserine-lactone synthase | | -0.54 | 9.5E-02 |
| PA3478 | RhlB | Rhamnosyltransferase chain B | | 0.71 | 1.8E-12 |
| PA3479 | RhlA | 3-(3-hydroxydecanoyloxy)decanoate synthase | | 0.70 | 2.3E-08 |
| PA3724 | LasB | Elastase (Neutral metalloproteinase) (PAE) (Pseudolysin) | | -0.80 | 1.1E-01 |
| **Phenazine biosynthesis** | | | | | |
| PA1899 | PhzA2 | Phenazine biosynthesis protein PhzA | | 1.94 | 1.2E-03 |
| PA1900 | PhzB2 | Phenazine biosynthesis protein PhzB | | 0.99 | 7.2E-03 |
| PA1904 | PhzF2 | Probable phenazine biosynthesis protein | | 0.61 | 2.2E-01 |
| PA1905 | PhzG2 | Pyridoxamine 5'-phosphate oxidase | | 1.22 | 2.1E-03 |
| PA4213 | PhzD1 | Phenazine biosynthesis protein PhzD | | 1.47 | 1.2E-10 |
| PA4214 | PhzE1 | Phenazine biosynthesis protein PhzE | | 0.95 | 1.3E-10 |
| **Carbon metabolism and amino acid metabolism** | | | | | |
| PA0400 | MetB | Probable cystathionine gamma-lyase | | 0.57 | 7.2E-07 |
| PA0792 | PrpD | Propionate catabolic protein | Fe | 0.95 | 1.1E-13 |
| PA0794 | | Probable aconitate hydratase | | -0.65 | 3.9E-08 |
| PA0865 | Hpd | 4-hydroxyphenylpyruvate dioxygenase (4HPPD) | | 0.56 | 2.4E-03 |

| | | | | | |
|---|---|---|---|---|---|
| PA1254 | LhpC | Probable dihydrodipicolinate synthetase | | -0.74 | 4.6E-04 |
| PA1255 | LhpK | Probable trans-3-hydroxy-L-proline dehydratase (T3LHyp dehydratase) | | -0.90 | 2.6E-02 |
| PA1260 | LhpP | Amino acid ABC transporter periplasmic binding protein | | -0.52 | 6.9E-02 |
| PA1261 | LhpR | Probable transcriptional regulator | | 0.68 | 3.9E-01 |
| PA1311 | PhnX | Phosphonoacetaldehyde hydrolase (Phosphonatase) | | -0.54 | 4.6E-01 |
| PA1422 | GbuR | GbuR | | 0.87 | 1.2E-01 |
| PA1562 | AcnA | Aconitate hydratase 1 | Fe | -0.98 | 6.2E-15 |
| PA2015 | LiuA | Putative isovaleryl-CoA dehydrogenase | | -0.78 | 2.1E-02 |
| PA2152 | | Probable trehalose synthase | | 0.54 | 2.0E-01 |
| PA2300 | ChiC | Chitinase | | 0.76 | 1.6E-05 |
| PA2416 | TreA | Periplasmic trehalase | | 0.81 | 4.2E-02 |
| PA3120 | LeuD | 3-isopropylmalate dehydratase small subunit | | -0.76 | 3.3E-03 |
| PA3121 | LeuC | 3-isopropylmalate dehydratase large subunit | Fe | -0.83 | 2.1E-07 |
| PA3236 | BetX | Probable glycine betaine-binding protein | | -0.68 | 5.0E-03 |
| PA3374 | PhnM | Amidohydro_3 domain-containing protein | | -0.94 | 9.0E-05 |
| PA3375 | PhnL | Probable ATP-binding component of ABC transporter | | -0.56 | 1.2E-01 |
| PA3376 | PhnK | Probable ATP-binding component of ABC transporter | | -0.60 | 4.2E-03 |
| PA3377 | PhnJ | Alpha-D-ribose 1-methylphosphonate 5-phosphate C-P lyase | | -0.76 | 6.4E-02 |
| PA3378 | PhnI | Uncharacterized protein | | -0.97 | 4.9E-03 |
| PA3379 | PhnH | Uncharacterized protein | | -0.80 | 6.3E-04 |
| PA3380 | PhnG | Uncharacterized protein | | -1.09 | 4.6E-03 |
| PA3417 | PdhA | Pyruvate dehydrogenase E1 component subunit alpha | | -0.54 | 9.5E-02 |
| PA3430 | | Putative aldolase class 2 protein PA3430 | | -0.86 | 3.5E-02 |
| PA3459 | | Probable glutamine amidotransferase | | -0.66 | 2.7E-06 |
| PA3506 | | Probable decarboxylase | Mn | -0.62 | 3.3E-01 |
| PA3524 | GloA1 | Lactoylglutathione lyase | Zn, Ni | -0.67 | 1.2E-02 |
| PA3896 | | Probable 2-hydroxyacid dehydrogenase | | 0.49 | 3.5E-01 |
| PA4150 | AcoA | Probable dehydrogenase E1 component | | -0.53 | 4.0E-01 |
| PA4151 | AcoB | Acetoin catabolism protein | | -0.72 | 2.5E-04 |
| PA4152 | AcoC | Probable hydrolase | | -0.67 | 5.0E-02 |
| PA4333 | FumA | Probable fumarase | Fe | -2.08 | 8.5E-13 |
| PA4470 | FumC1 | Fumarate hydratase | Mn | 3.11 | 8.0E-16 |
| PA4628 | LysP | Lysine-specific permease | | 0.65 | 2.1E-01 |
| PA5354 | GlcE | Glycolate oxidase subunit GlcE | | -0.61 | 2.2E-01 |
| PA5376 | CbcV | | | -0.53 | 4.7E-01 |
| PA5398 | DgcA | Dimethylglycine catabolism | | -1.40 | 9.0E-05 |
| PA5410 | GbcA | Glycine betaine catabolism protein | Fe | -1.41 | 1.3E-07 |
| PA5415 | GlyA1 | Serine hydroxymethyltransferase | | -0.79 | 2.3E-06 |
| PA5416 | SoxB | Sarcosine oxidase beta subunit | | -0.69 | 2.7E-01 |
| PA5417 | SoxD | Sarcosine oxidase delta subunit | | -0.66 | 2.0E-02 |
| PA5418 | SoxA | Sarcosine oxidase alpha subunit | Fe | -0.60 | 5.7E-03 |
| PA5421 | FdhA | Glutathione-independent formaldehyde dehydrogenase (FALDH) (FDH) | Zn | -0.61 | 7.3E-09 |
| PA5445 | PsecoA | Probable coenzyme A transferase | | -0.62 | 3.2E-01 |

| | | | | | |
|---|---|---|---|---|---|
| **Sulfur assimilation** | | | | | |
| PA0280 | CysA | Sulfate transport protein | | 1.17 | 1.2E-06 |
| PA0282 | CysT | Sulfate transport protein | | 1.28 | 5.5E-06 |
| PA0283 | Sbp | Sulfate-binding protein precursor | | 2.05 | 2.8E-19 |
| PA0500 | BioB | Biotin synthase | Fe | 0.79 | 3.4E-02 |
| PA0916 | YliG | Ribosomal protein S12 methylthiotransferase | Fe | -0.85 | 8.9E-02 |
| PA1192 | YdaO | tRNA-cytidine(32) 2-sulfurtransferase | Fe | -0.78 | 1.8E-01 |
| PA1505 | MoaA2 | Molybdenum cofactor biosynthesis protein A 2 | Fe | -0.61 | 3.7E-01 |
| PA1838 | CysI | Sulfite reductase | Fe | 0.68 | 2.0E-14 |
| PA2062 | | Probable pyridoxal-phosphate dependent enzyme | Fe | 3.05 | 3.9E-05 |
| PA2566 | | Conserved hypothetical protein | | -0.75 | 1.7E-01 |
| PA2594 | | Conserved hypothetical protein | | 1.54 | 4.0E-05 |
| PA3445 | | Conserved hypothetical protein | | 2.87 | 9.1E-06 |
| PA3938 | TauA | Probable periplasmic taurine-binding protein | | 2.47 | 4.1E-04 |
| PA3980 | MiaB | tRNA-2-methylthio-N(6)-dimethylallyladenosine synthase | Fe | -1.74 | 5.8E-06 |
| PA3996 | LipA | Lipoate synthase (Sulfur insertion protein LipA) | Fe | -0.63 | 1.7E-01 |
| PA4442 | CysN | ATP sulfurylase GTP-binding subunit/APS kinase | | 0.83 | 5.3E-10 |
| PA4443 | CysD | ATP sulfurylase small subunit | | 1.03 | 3.9E-03 |
| PA4973 | ThiC | Phosphomethylpyrimidine synthase | Fe | -0.56 | 4.0E-02 |
| PA5025 | MetY | Homocysteine synthase | | 0.86 | 9.1E-03 |
| **Respiration** | | | | | |
| PA0527 | Dnr | Transcriptional regulator | | -0.94 | 1.5E-02 |
| PA1173 | NapB | Periplasmic nitrate reductase | Fe | -0.99 | 7.1E-03 |
| PA1174 | NapA | Periplasmic nitrate reductase | Fe | -1.31 | 1.2E-12 |
| PA1175 | NapD | NapA signal peptide-binding chaperone | | -0.72 | 1.4E-02 |
| PA1176 | NapF | Ferredoxin-type protein | Fe | -0.88 | 2.0E-02 |
| PA1544 | Anr | Transcriptional activator protein | Fe | 0.69 | 3.4E-05 |
| PA2266 | | Probable cytochrome c | Fe | -0.56 | 4.6E-01 |
| PA3331 | | Cytochrome P450 | Fe | -0.67 | 7.6E-04 |
| PA3872 | NarI | Respiratory nitrate reductase gamma chain | Fe | -0.58 | 3.6E-02 |
| PA4133 | CcoN | Cytochrome c oxidase subunit (Cbb3-type) | Fe | -1.11 | 1.2E-02 |
| **Anr regulon** | | | | | |
| PA1546 | HemN | Oxygen-independent coproporphyrinogen III oxidase (CPO) | Fe | -1.63 | 2.6E-06 |
| PA1673 | | Bacteriohemerythrin | Fe | -0.95 | 5.3E-06 |
| PA3049 | Rmf | Ribosome modulation factor | | 0.74 | 3.8E-03 |
| PA3126 | IbpA | Heat-shock protein IbpA | | -0.60 | 2.5E-04 |
| PA3572 | | Hypothetical protein | | -0.52 | 2.5E-03 |
| PA3919 | YlaK | PINc domain-containing protein | | -0.77 | 5.5E-02 |
| PA4587 | CcpR | Cytochrome c551 peroxidase | Fe | -0.52 | 1.3E-04 |
| PA4880 | | Probable bacterioferritin | Fe | -2.95 | 8.1E-16 |
| PA5475 | | Acetyltransferase | | -0.61 | 4.1E-02 |
| **Oxidative stress regulation** | | | | | |

| | | | | | |
|---|---|---|---|---|---|
| PA0140 | AhpF | Alkyl hydroperoxide reductase subunit F | | -0.60 | 2.1E-06 |
| PA0849 | TrxB2 | Thioredoxin reductase | | -0.77 | 4.2E-04 |
| PA2185 | KatN | Non-heme catalase KatN | Mn | -0.91 | 1.2E-01 |
| PA2580 | MdaB | Flavodoxin_2 domain-containing protein | | -0.55 | 1.0E-02 |
| PA3450 | LsfA | Probable antioxidant protein | | 2.49 | 1.2E-21 |
| PA3533 | GrxD | Glutaredoxin | | -0.81 | 3.6E-08 |
| PA4236 | KatA | Catalase | Fe | -1.08 | 1.4E-08 |
| PA4366 | SodB | Superoxide dismutase [Fe] | Fe | -0.96 | 7.1E-03 |
| PA4468 | SodA | Superoxide dismutase [Mn] | Mn | 2.81 | 2.6E-14 |
| **Nucleotide synthesis and metabolism** | | | | | |
| PA0441 | Dht | D-hydantoinase/dihydropyrimidinase (DHPase) | Zn | -0.71 | 2.2E-02 |
| PA1155 | NrdB | Ribonucleoside-diphosphate reductase subunit beta | Fe | -0.61 | 2.0E-02 |
| PA1156 | NrdA | Ribonucleoside-diphosphate reductase | | -0.62 | 1.9E-06 |
| PA1932 | | Probable hydroxylase molybdopterin-containing subunit | | -0.89 | 9.0E-02 |
| **Lipid metabolism** | | | | | |
| PA0347 | GlpQ | Glycerophosphoryl diester phosphodiesterase, periplasmic | | -1.06 | 2.6E-11 |
| PA2862 | LipA | Triacylglycerol lipase | | -0.52 | 2.1E-01 |
| PA3092 | FadH1 | 2,4-dienoyl-CoA reductase FadH1 | | -1.11 | 2.0E-04 |
| PA3319 | PlcN | Non-hemolytic phospholipase C (PLC-N) | | -0.84 | 3.6E-14 |
| PA3333 | FabH | 3-oxoacyl-[acyl-carrier-protein] synthase 3 | | -0.85 | 4.6E-06 |
| PA4350 | OlsB | Uncharacterized protein | | -0.55 | 5.0E-02 |
| PA4353 | YajB | Uncharacterized protein | | -0.56 | 6.0E-03 |
| PA4661 | PagL | Lipid A deacylase PagL | | 0.51 | 6.1E-02 |
| **Secreted proteins and secretion systems** | | | | | |
| PA0572 | ImpA | Immunomodulating metalloprotease | Zn | 0.71 | 1.1E-02 |
| PA0688 | PhoA | Alkaline phosphatase L (L-AP) | | -0.74 | 5.8E-09 |
| PA1245 | AprX | Uncharacterized protein | | 3.02 | 3.9E-10 |
| PA1246 | AprD | Alkaline protease secretion ATP-binding protein AprD | | 2.25 | 2.2E-09 |
| PA1247 | AprE | Alkaline protease secretion protein AprE | | 2.11 | 3.1E-03 |
| PA1249 | AprA | Serralysin (Alkaline metalloproteinase) (AP) | Zn | 1.82 | 1.4E-10 |
| PA1250 | AprI | Proteinase inhibitor (Aprin) | Zn | 0.96 | 1.0E-03 |
| PA3099 | XcpV | Type II secretion system protein I | | 0.79 | 1.4E-01 |
| **Oxidation-reduction processes** | | | | | |
| PA0785 | AzoR1 | FMN-dependent NADH-azoreductase 1 | | -0.69 | 2.3E-01 |
| PA0840 | | Probable oxidoreductase | | -0.51 | 1.3E-02 |
| PA2033 | | FAD-binding FR-type domain-containing protein | | 1.72 | 5.8E-10 |
| PA2158 | | Probable alcohol dehydrogenase (Zn-dependent) | Zn | 0.75 | 1.4E-01 |
| PA2378 | | Probable aldehyde dehydrogenase | | -0.93 | 8.1E-05 |
| PA2379 | | Probable oxidoreductase | Fe | -1.03 | 1.4E-03 |
| PA5150 | | Probable short-chain dehydrogenase | | 1.45 | 1.0E-09 |
| PA5187 | | Probable acyl-CoA dehydrogenase | | -0.61 | 2.8E-01 |

| | | | | | |
|---|---|---|---|---|---|
| PA5188 | | Probable 3-hydroxyacyl-CoA dehydrogenase | | -0.63 | 1.4E-02 |
| **Other cellular transporters** | | | | | |
| PA2203 | | Probable amino acid permease | | 1.74 | 6.8E-03 |
| PA2491 | MexS | Probable oxidoreductase | | -1.06 | 1.2E-09 |
| PA2493 | MexE | Resistance-Nodulation-Cell Division (RND) multidrug efflux membrane fusion protein | | -0.81 | 2.3E-07 |
| PA2494 | MexF | Efflux pump membrane transporter | | -0.75 | 3.2E-07 |
| PA3280 | OprO | Porin O | | -1.02 | 8.4E-07 |
| PA3931 | | Uncharacterized protein | | 1.54 | 1.8E-22 |
| PA5103 | PuuR | OpuAC domain-containing protein | | 0.71 | 1.2E-01 |
| PA5217 | | Probable binding protein component of ABC iron transporter | | 0.50 | 8.7E-02 |
| **Others** | | | | | |
| PA0168 | YigZ | Uncharacterized protein | | 0.56 | 2.9E-01 |
| PA0225 | | Probable transcriptional regulator | | 0.78 | 1.0E-01 |
| PA0277 | | Peptidase_M48 domain-containing protein | | -0.58 | 3.6E-02 |
| PA0284 | | Uncharacterized protein | | 2.35 | 3.5E-07 |
| PA0312 | | DUF523 domain-containing protein | | -1.44 | 3.1E-02 |
| PA0321 | AphB | Acetylpolyamine amidohydrolase 2 | | -0.53 | 1.7E-03 |
| PA0423 | PasP | UPF0312 protein PA0423 | | 0.61 | 3.8E-06 |
| PA0540 | | Uncharacterized protein | | 0.82 | 6.5E-03 |
| PA0622 | | Probable bacteriophage protein | | 0.62 | 1.3E-03 |
| PA0623 | | Probable bacteriophage protein | | 0.68 | 1.0E-02 |
| PA0633 | | Uncharacterized protein | | 1.19 | 3.0E-02 |
| PA0634 | | Uncharacterized protein | | 0.52 | 2.8E-02 |
| PA0641 | | Probable bacteriophage protein | | 0.96 | 4.1E-02 |
| PA0757 | | Probable two-component sensor | | 0.58 | 5.6E-01 |
| PA0807 | AmpDh3 | AmpDh3 | | 0.51 | 1.6E-03 |
| PA0929 | PirR | Two-component response regulator | | -1.24 | 6.0E-05 |
| PA0981 | | Uncharacterized protein | | -0.97 | 9.2E-05 |
| PA0984 | | Colicin immunity protein | | 0.52 | 1.0E-01 |
| PA0985 | PyoS5 | Pyocin S5 | | 0.91 | 6.7E-07 |
| PA1017 | PauA | Pimeloyl-CoA synthetase | | 1.23 | 5.2E-01 |
| PA1026 | | Uncharacterized protein | | -0.80 | 4.2E-02 |
| PA1484 | | Probable transcriptional regulator | | 0.62 | 1.3E-01 |
| PA1522 | | Uncharacterized protein | | -0.55 | 1.5E-01 |
| PA1727 | MucR | Uncharacterized signaling protein PA1727 | | -0.71 | 5.7E-02 |
| PA1784 | | Alginate_lyase2 domain-containing protein | | 1.36 | 1.9E-02 |
| PA1837 | | Uncharacterized protein | | 1.10 | 6.2E-02 |
| PA2034 | | Methyltransf_25 domain-containing protein | | 2.07 | 1.3E-09 |
| PA2048 | | ABM domain-containing protein | | -0.71 | 1.0E-03 |
| PA2066 | | DUF218 domain-containing protein | | 0.99 | 3.2E-02 |
| PA2086 | | Probable epoxide hydrolase | | 3.48 | 1.6E-17 |
| PA2161 | | Uncharacterized protein | | 0.62 | 3.7E-01 |
| PA2171 | | Hemerythrin domain-containing protein | | -2.46 | 1.4E-01 |

| | | | | | |
|---|---|---|---|---|---|
| PA2184 | YciE | DUF892 domain-containing protein | | 0.63 | 1.3E-03 |
| PA2204 | | Probable binding protein component of ABC transporter | | 1.82 | 4.9E-23 |
| PA2309 | | Uncharacterized protein | | 1.50 | 9.9E-04 |
| PA2328 | | Uncharacterized protein | | 1.15 | 1.8E-05 |
| PA2384 | | Uncharacterized protein | | 2.81 | 2.6E-12 |
| PA2414 | | L-sorbosone dehydrogenase | | 0.75 | 1.1E-01 |
| PA2448 | | Amidohydro_3 domain-containing protein | | 0.60 | 2.6E-04 |
| PA2531 | | Probable aminotransferase | | 2.63 | 1.7E-07 |
| PA2535 | | Probable oxidoreductase | | -0.53 | 3.6E-08 |
| PA2599 | | PBPb domain-containing protein | | 0.79 | 2.7E-02 |
| PA2603 | | Probable thiosulfate sulfurtransferase | | 0.58 | 2.5E-02 |
| PA2635 | | Uncharacterized protein | | -1.18 | 2.3E-04 |
| PA2765 | | Uncharacterized protein | Fe | -0.92 | 3.2E-09 |
| PA2786 | | GAF domain-containing protein | | 3.91 | 1.2E-17 |
| PA2804 | | HD_domain domain-containing protein | | -1.16 | 1.1E-05 |
| PA2821 | | Probable glutathione S-transferase | | -1.06 | 4.5E-07 |
| PA2831 | | Uncharacterized protein | Zn | -0.57 | 3.7E-08 |
| PA2915 | | Lactamase_B domain-containing protein | | -0.64 | 1.2E-02 |
| PA2956 | | Elp3 domain-containing protein | Fe | -0.97 | 7.2E-02 |
| PA3009 | | Uncharacterized protein | | -0.76 | 8.7E-02 |
| PA3010 | | Uncharacterized protein | | -0.54 | 8.3E-03 |
| PA3022 | | MOSC domain-containing protein | Mo | -0.63 | 6.0E-03 |
| PA3067 | | Probable transcriptional regulator | | -1.20 | 3.2E-01 |
| PA3124 | | Probable transcriptional regulator | | -1.20 | 6.7E-02 |
| PA3219 | | Metallophos_2 domain-containing protein | | -0.98 | 2.0E-08 |
| PA3240 | | Putative quercetin 2,3-dioxygenase PA3240 (Putative quercetinase) | | -0.63 | 1.0E-01 |
| PA3277 | | Probable short-chain dehydrogenase | | 0.57 | 2.4E-01 |
| PA3321 | | Probable transcriptional regulator | | -0.82 | 4.2E-02 |
| PA3327 | | Probable non-ribosomal peptide synthetase | | -1.48 | 2.3E-04 |
| PA3328 | | Probable FAD-dependent monooxygenase | | -1.01 | 1.1E-02 |
| PA3329 | | Condensation domain-containing protein | | -0.79 | 6.8E-09 |
| PA3330 | | Probable short chain dehydrogenase | | -1.20 | 1.8E-05 |
| PA3332 | | Uncharacterized PhzA/B-like protein PA3332 | | -0.64 | 2.4E-02 |
| PA3441 | SsuF | Probable molybdopterin-binding protein | | 3.04 | 3.2E-09 |
| PA3815 | IscR | HTH-type transcriptional regulator IscR | | -0.56 | 8.9E-02 |
| PA3844 | | GST N-terminal domain-containing protein | | -0.88 | 6.7E-09 |
| PA3846 | | Isochorismatase domain-containing protein | | -0.95 | 3.9E-03 |
| PA3865.1 | | Pyocin S4 immunity protein | | 2.14 | 2.8E-02 |
| PA3866 | | Pyocin protein | | 1.67 | 2.2E-12 |
| PA3932 | | Probable transcriptional regulator | | 2.24 | 6.1E-07 |
| PA4131 | | Probable iron-sulfur protein | Fe | -1.29 | 3.8E-03 |
| PA4165 | | Probable transcriptional regulator | | 0.60 | 3.0E-02 |
| PA4195 | | Probable binding protein component of ABC transporter | | 2.01 | 2.0E-03 |
| PA4197 | BfiS | | | -1.20 | 1.8E-01 |
| PA4325 | | DUF4124 domain-containing protein | | -0.87 | 2.7E-01 |

| | | | | | |
|---|---|---|---|---|---|
| PA4370 | IcmP | Insulin-cleaving metalloproteinase outer membrane protein | | 1.12 | 1.1E-14 |
| PA4371 | | Uncharacterized protein | Fe | 0.64 | 2.8E-08 |
| PA4373 | | Uncharacterized protein | | 0.73 | 2.4E-01 |
| PA4384 | | Uncharacterized protein | | 0.50 | 1.6E-01 |
| PA4390 | | Uncharacterized protein | | 1.05 | 1.3E-01 |
| PA4573 | | Uncharacterized protein | | 1.95 | 1.9E-01 |
| PA4664 | PrmC | S-adenosylmethionine-dependent methyltransferase | | -0.75 | 1.2E-02 |
| PA4674 | VapI | Antotoxin HigA | | -0.64 | 1.6E-01 |
| PA4692 | MsrP | Protein-methionine-sulfoxide reductase catalytic subunit MsrP | | -0.71 | 1.1E-01 |
| PA4704 | CbpA | cAMP-binding protein A | | -0.55 | 2.9E-01 |
| PA4728 | FolK | 2-amino-4-hydroxy-6-hydroxymethyldihydropteridine pyrophosphokinase | | 0.96 | 5.4E-02 |
| PA4792 | | GP-PDE domain-containing protein | | 1.61 | 1.2E-11 |
| PA4875 | | MOSC domain-containing protein | Mo | -1.06 | 1.7E-04 |
| PA4928 | YgiR | UPF0313 protein | Fe | -1.31 | 6.7E-03 |
| PA4943 | HflX | GTPase HflX (GTP-binding protein HflX) | | -0.55 | 3.3E-01 |
| PA5081 | | Nudix hydrolase domain-containing protein | | 0.69 | 3.1E-02 |
| PA5189 | | Probable transcriptional regulator | | 0.50 | 4.4E-01 |
| PA5506 | | Uncharacterized protein | | -0.66 | 2.2E-01 |

Proteins exhibiting significant abundance differences between wt and Δ*bfd* cells were functionally categorized using the Kyoto Encyclopedia of Genes and Genomes (KEGG) Orthology classification [1], the Pseudomonas Genome Database [2], and the UniProt database [3].

**Table S2**: Proteins exhibiting significant abundance differences between wt and Δ*bfd* cells are compared to proteins exhibiting significant abundance differences between wt cells cultured in high vs. low iron conditions

| Protein ID | Name | Function | Metal binding | This study FC = Δ*bfd*/wt | | Low *vs* High Fe ref [4] | |
|---|---|---|---|---|---|---|---|
| | | | | *log*$_2$FC | *p* value | *log*$_2$FC | *p* value |
| **Pyoverdine biosynthesis** | | | | | | | |
| PA2385 | PvdQ | Acyl-homoserine lactone acylase | | 3.14 | 3.9E-10 | 2.67 | 1.5E-05 |
| PA2386 | PvdA | L-ornithine N(5)-monooxygenase | | 3.02 | 1.2E-15 | 3.00 | 1.2E-05 |
| PA2388 | FpvR | | | -2.18 | 1.7E-03 | ND | |
| PA2389 | PvdR | | | 2.81 | 2.8E-06 | 2.07 | 3.6E-05 |
| PA2390 | PvdT | Macrolide export ATP-binding/permease protein | | 2.73 | 2.0E-10 | 2.18 | 1.9E-04 |
| PA2391 | OpmQ | Probable outer membrane protein | | 2.00 | 1.5E-04 | 1.77 | 6.2E-04 |
| PA2392 | PvdP | | | 4.11 | 5.2E-10 | 2.67 | 6.6E-05 |
| PA2393 | PvdM | Probable dipeptidase | | 3.22 | 1.3E-13 | 3.05 | 9.0E-06 |
| PA2394 | PvdN | | | 3.61 | 2.3E-11 | 3.15 | 1.0E-06 |
| PA2395 | PvdO | | | 3.48 | 7.9E-16 | 1.76 | 4.6E-03 |
| PA2396 | PvdF | Pyoverdine synthetase F | | 3.25 | 1.1E-10 | 2.30 | 1.8E-04 |
| PA2397 | PvdE | Pyoverdine biosynthesis protein | | 3.16 | 1.6E-12 | 2.98 | 3.1E-05 |
| PA2398 | FpvA | Ferripyoverdine receptor | | 1.72 | 3.3E-07 | 3.83 | 1.8E-07 |
| PA2399 | PvdD | Pyoverdine synthetase D | | 2.75 | 1.8E-13 | ND | |
| PA2400 | PvdJ | Probable non-ribosomal peptide synthetase | | 2.87 | 1.4E-12 | 1.22 | 7.1E-04 |
| PA2402 | PvdI | Pyoverdine peptide synthetase | | 2.87 | 4.6E-18 | ND | |
| PA2403 | FpvG | Uncharacterized protein | | 2.90 | 6.8E-12 | 0.47 | 3.4E-01 |
| PA2404 | FpvH | Uncharacterized protein | | 2.52 | 1.8E-04 | 4.38 | 7.9E-06 |
| PA2405 | FpvJ | Probable adhesion protein | | 2.94 | 1.6E-11 | ND | |
| PA2407 | FpvC | Probable ATP-binding component of ABC transporter | | 2.97 | 1.6E-09 | 1.97 | 9.9E-04 |
| PA2408 | FpvD | Uncharacterized protein | | 1.20 | 1.1E-03 | infinity | 1.9E-07 |
| PA2410 | FpvF | Probable thioesterase | | 2.44 | 1.9E-09 | 2.62 | 4.5E-04 |
| PA2411 | | MbtH domain-containing protein | | 2.73 | 6.3E-10 | 1.97 | 2.1E-04 |
| PA2412 | | L-2,4-diaminobutyrate:2-ketoglutarate 4-aminotransferase | | 1.20 | 4.5E-03 | 3.66 | 5.8E-05 |
| PA2413 | PvdH | L-sorbosone dehydrogenase | | 3.10 | 6.2E-19 | 3.40 | 1.9E-05 |
| PA2424 | PvdL | | | 2.76 | 6.2E-20 | ND | |
| PA2425 | PvdG | | | 1.75 | 6.3E-05 | ND | |
| PA2426 | PvdS | Sigma factor | | 0.91 | 4.8E-03 | 1.70 | 4.5E-04 |
| PA4168 | FpvB | Second ferric pyoverdine receptor FpvB | | 0.67 | 2.9E-01 | 2.56 | 5.2E-06 |
| **Pyochelin biosynthesis** | | | | | | | |
| PA4218 | FptX | Probable transporter | | 3.14 | 2.1E-08 | 2.71 | 9.9E-05 |
| PA4219 | YfpB | Uncharacterized protein | | 2.83 | 9.1E-11 | ND | |
| PA4221 | FptA | Fe(3+)-pyochelin receptor (Fe(III)-pyochelin receptor) | | 2.50 | 2.9E-10 | 4.07 | 3.5E-06 |
| PA4222 | PchI | Probable ATP-binding component of ABC transporter | | 3.36 | 8.1E-19 | 2.49 | 4.1E-04 |

| | | | | | | | |
|---|---|---|---|---|---|---|---|
| PA4223 | PchH | Probable ATP-binding component of ABC transporter | | 3.32 | 1.8E-11 | 2.71 | 1.0E-04 |
| PA4224 | PchG | Pyochelin biosynthetic protein | | 2.74 | 3.2E-21 | 2.00 | 5.9E-03 |
| PA4225 | PchF | Pyochelin synthetase | | 2.38 | 9.0E-23 | 0.87 | 8.4E-03 |
| PA4226 | PchE | Dihydroaeruginoic acid synthetase | | 2.79 | 9.9E-17 | 2.60 | 9.7E-04 |
| PA4227 | PchR | Regulatory protein | | 1.19 | 4.1E-06 | 2.93 | 3.1E-04 |
| PA4228 | PchD | Pyochelin biosynthesis protein | | 2.84 | 3.4E-17 | 1.83 | 1.0E-03 |
| PA4229 | PchC | Pyochelin biosynthetic protein | | 1.14 | 1.0E-07 | ND | |
| PA4230 | PchB | Isochorismate pyruvate lyase | | 3.10 | 6.1E-18 | 4.50 | 1.8E-04 |
| PA4231 | PchA | Salicylate biosynthesis isochorismate synthase | | 1.93 | 1.2E-05 | ND | |
| **Heme iron acquisition** | | | | | | | |
| PA0672 | HemO | Heme oxygenase | | 1.49 | 6.9E-13 | 2.51 | 1.0E-04 |
| PA4708 | PhuT | Heme-transport protein | | 1.42 | 2.3E-04 | 2.62 | 4.4E-06 |
| PA4709 | PhuS | | | 1.23 | 3.6E-04 | 2.34 | 4.1E-06 |
| PA4710 | PhuR | Heme/Hemoglobin uptake outer membrane receptor | | 2.59 | 1.5E-14 | 3.35 | 1.4E-06 |
| **Other iron acquisition and transport related proteins** | | | | | | | |
| PA0471 | FiuR | Probable transmembrane sensor | | 0.66 | 5.0E-02 | 2.52 | 3.8E-06 |
| PA2466 | FoxA | Ferrioxamine receptor FoxA | | 0.56 | 7.5E-04 | | |
| PA3901 | FecA | Fe(III) dicitrate transport protein FecA | Fe | 0.81 | 1.2E-01 | 2.87 | 3.3E-05 |
| PA4514 | PiuA | Probable outer membrane receptor for iron transport | | 1.02 | 1.6E-04 | 1.28 | 1.5E-04 |
| PA4675 | OptH | Probable TonB-dependent receptor | | 0.53 | 4.8E-01 | | |
| **Quorum sensing** | | | | | | | |
| PA0996 | PqsA | Anthranilate--CoA ligase | | 1.71 | 1.4E-03 | | |
| PA0997 | PqsB | Hypothetical protein | | 2.06 | 1.6E-06 | | |
| PA0998 | PqsC | Hypothetical protein | | 2.28 | 1.1E-04 | | |
| PA0999 | PqsD | 3-oxoacyl-ACP synthase | | 2.39 | 7.2E-11 | 1.42 | 5.2E-05 |
| PA1000 | PqsE | Thioesterase PqsE | | 2.08 | 9.6E-03 | | |
| PA1871 | LasA | Protease LasA (Staphylolytic protease) | | 0.55 | 8.2E-02 | | |
| PA2569 | | Uncharacterized protein | | 0.78 | 4.3E-02 | | |
| PA2570 | LecA | PA-I galactophilic lectin (PA-IL) (Galactose-binding lectin) | | 1.53 | 1.3E-02 | | |
| PA3476 | RhlI | Acyl-homoserine-lactone synthase | | -0.54 | 9.5E-02 | -1.08 | 2.8E-03 |
| PA3478 | RhlB | Rhamnosyltransferase chain B | | 0.71 | 1.8E-12 | | |
| PA3479 | RhlA | 3-(3-hydroxydecanoyloxy) decanoate synthase | | 0.70 | 2.3E-08 | | |
| PA3724 | LasB | Elastase (Neutral metalloproteinase) (PAE) (Pseudolysin) | | -0.80 | 1.1E-01 | 1.01 | 2.2E-02 |
| **Phenazine biosynthesis** | | | | | | | |
| PA1899 | PhzA2 | Phenazine biosynthesis protein | | 1.94 | 1.2E-03 | | |
| PA1900 | PhzB2 | Phenazine biosynthesis protein | | 0.99 | 7.2E-03 | -2.66 | 7.6E-05 |
| PA1904 | PhzF2 | Probable phenazine biosynthesis protein | | 0.61 | 2.2E-01 | -1.15 | 9.6E-04 |
| PA1905 | PhzG2 | Pyridoxamine 5'-phosphate oxidase | | 1.22 | 2.1E-03 | | |

| | | | | | | | | |
|---|---|---|---|---|---|---|---|---|
| PA4213 | PhzD1 | Phenazine biosynthesis protein | | 1.47 | 1.2E-10 | -1.91 | 3.2E-03 |
| PA4214 | PhzE1 | Phenazine biosynthesis protein | | 0.95 | 1.3E-10 | | |
| **Carbon metabolism and amino acid metabolism** | | | | | | | |
| PA0400 | MetB | Probable cystathionine gamma-lyase | | 0.57 | 7.2E-07 | 1.66 | 2.0E-04 |
| PA0792 | PrpD | Propionate catabolic protein | Fe | 0.95 | 1.1E-13 | 2.10 | 7.5E-06 |
| PA0794 | | Probable aconitate hydratase | | -0.65 | 3.9E-08 | -2.13 | 1.9E-05 |
| PA0865 | Hpd | 4-hydroxyphenylpyruvate dioxygenase (4HPPD) | | 0.56 | 2.4E-03 | 1.61 | 1.1E-03 |
| PA1254 | LhpC | Probable dihydrodipicolinate synthetase | | -0.74 | 4.6E-04 | | |
| PA1255 | LhpK | Probable trans-3-hydroxy-L-proline dehydratase (T3LHyp dehydratase) | | -0.90 | 2.6E-02 | | |
| PA1260 | LhpP | Amino acid ABC transporter Periplasmic binding protein | | -0.52 | 6.9E-02 | | |
| PA1261 | LhpR | Probable transcriptional regulator | | 0.68 | 3.9E-01 | | |
| PA1311 | PhnX | Phosphonoacetaldehyde hydrolase (Phosphonatase) | | -0.54 | 4.6E-01 | | |
| PA1422 | GbuR | GbuR | | 0.87 | 1.2E-01 | | |
| PA1562 | AcnA | Aconitate hydratase 1 | Fe | -0.98 | 6.2E-15 | -2.59 | 3.5E-05 |
| PA2015 | LiuA | Putative isovaleryl-CoA dehydrogenase | | -0.78 | 2.1E-02 | 1.37 | 4.0E-05 |
| PA2152 | | Probable trehalose synthase | | 0.54 | 2.0E-01 | | |
| PA2300 | ChiC | Chitinase | | 0.76 | 1.6E-05 | | |
| PA2416 | TreA | Periplasmic trehalase | | 0.81 | 4.2E-02 | | |
| PA3120 | LeuD | 3-isopropylmalate dehydratase small subunit | | -0.76 | 3.3E-03 | | |
| PA3121 | LeuC | 3-isopropylmalate dehydratase large subunit | Fe | -0.83 | 2.1E-07 | -1.94 | 2.2E-04 |
| PA3236 | BetX | Probable glycine betaine-binding protein | | -0.68 | 5.0E-03 | | |
| PA3374 | PhnM | Amidohydro_3 domain-containing protein | | -0.94 | 9.0E-05 | | |
| PA3375 | PhnL | Probable ATP-binding component of ABC transporter | | -0.56 | 1.2E-01 | | |
| PA3376 | PhnK | Probable ATP-binding component of ABC transporter | | -0.60 | 4.2E-03 | | |
| PA3377 | PhnJ | Alpha-D-ribose 1-methylphosphonate 5-phosphate C-P lyase | | -0.76 | 6.4E-02 | | |
| PA3378 | PhnI | Uncharacterized protein | | -0.97 | 4.9E-03 | | |
| PA3379 | PhnH | Uncharacterized protein | | -0.80 | 6.3E-04 | | |
| PA3380 | PhnG | Uncharacterized protein | | -1.09 | 4.6E-03 | | |
| PA3417 | PdhA | Pyruvate dehydrogenase E1 component subunit alpha | | -0.54 | 9.5E-02 | | |
| PA3430 | | Putative aldolase class 2 protein | | -0.86 | 3.5E-02 | -8.45 | 6.2E-04 |
| PA3459 | | Probable glutamine amidotransferase | | -0.66 | 2.7E-06 | | |
| PA3506 | | Probable decarboxylase | Mn | -0.62 | 3.3E-01 | | |
| PA3524 | GloA1 | Lactoylglutathione lyase | Zn, Ni | -0.67 | 1.2E-02 | -1.73 | 7.7E-04 |
| PA3896 | | Probable 2-hydroxyacid dehydrogenase | | 0.49 | 3.5E-01 | | |
| PA4150 | AcoA | Probable dehydrogenase E1 component | | -0.53 | 4.0E-01 | | |
| PA4151 | AcoB | Acetoin catabolism protein | | -0.72 | 2.5E-04 | | |
| PA4152 | AcoC | Probable hydrolase | | -0.67 | 5.0E-02 | | |
| PA4333 | FumA | Probable fumarase | Fe | -2.08 | 8.5E-13 | -3.96 | 7.2E-06 |
| PA4470 | FumC1 | Fumarate hydratase | Mn | 3.11 | 8.0E-16 | 4.95 | 3.2E-08 |
| PA4628 | LysP | Lysine-specific permease | | 0.65 | 2.1E-01 | | |

| | | | | | | | |
|---|---|---|---|---|---|---|---|
| PA5354 | GlcE | Glycolate oxidase subunit GlcE | | -0.61 | 2.2E-01 | | |
| PA5376 | CbcV | | | -0.53 | 4.7E-01 | | |
| PA5398 | DgcA | Dimethylglycine catabolism | | -1.40 | 9.0E-05 | 1.31 | 1.2E-03 |
| PA5410 | GbcA | glycine betaine catabolism protein | Fe | -1.41 | 1.3E-07 | | |
| PA5415 | GlyA1 | Serine hydroxymethyltransferase | | -0.79 | 2.3E-06 | | |
| PA5416 | SoxB | Sarcosine oxidase beta subunit | | -0.69 | 2.7E-01 | | |
| PA5417 | SoxD | Sarcosine oxidase delta subunit | | -0.66 | 2.0E-02 | | |
| PA5418 | SoxA | Sarcosine oxidase alpha subunit | Fe | -0.60 | 5.7E-03 | | |
| PA5421 | FdhA | Glutathione-independent formaldehyde dehydrogenase (FALDH) (FDH) | Zn | -0.61 | 7.3E-09 | | |
| PA5445 | PsecoA | Probable coenzyme A transferase | | -0.62 | 3.2E-01 | | |
| **Sulfur assimilation** | | | | | | | |
| PA0280 | CysA | Sulfate transport protein | | 1.17 | 1.2E-06 | | |
| PA0282 | CysT | Sulfate transport protein | | 1.28 | 5.5E-06 | | |
| PA0283 | Sbp | Sulfate-binding protein precursor | | 2.05 | 2.8E-19 | | |
| PA0500 | BioB | Biotin synthase | Fe | 0.79 | 3.4E-02 | -0.61 | 4.7E-03 |
| PA0916 | YliG | Ribosomal protein S12 methylthiotransferase | Fe | -0.85 | 8.9E-02 | 1.11 | 1.8E-03 |
| PA1192 | YdaO | tRNA-cytidine(32) 2-sulfurtransferase | Fe | -0.78 | 1.8E-01 | | |
| PA1505 | MoaA2 | Molybdenum cofactor biosynthesis protein A 2 | Fe | -0.61 | 3.7E-01 | | |
| PA1838 | CysI | Sulfite reductase | Fe | 0.68 | 2.0E-14 | -1.46 | 5.6E-05 |
| PA2062 | | Probable pyridoxal-phosphate dependent enzyme | Fe | 3.05 | 3.9E-05 | | |
| PA2566 | | Conserved hypothetical protein | | -0.75 | 1.7E-01 | | |
| PA2594 | | Conserved hypothetical protein | | 1.54 | 4.0E-05 | | |
| PA3445 | | Conserved hypothetical protein | | 2.87 | 9.1E-06 | -1.87 | 3.6E-02 |
| PA3938 | TauA | Probable periplasmic taurine-binding protein | | 2.47 | 4.1E-04 | | |
| PA3980 | MiaB | tRNA-2-methylthio-N(6)-dimethylallyladenosine synthase | Fe | -1.74 | 5.8E-06 | | |
| PA3996 | LipA | Lipoate synthase (Sulfur insertion protein LipA) | Fe | -0.63 | 1.7E-01 | | |
| PA4442 | CysN | ATP sulfurylase GTP-binding subunit/APS kinase | | 0.83 | 5.3E-10 | -1.60 | 2.7E-04 |
| PA4443 | CysD | ATP sulfurylase small subunit | | 1.03 | 3.9E-03 | -2.09 | 6.1E-06 |
| PA4973 | ThiC | Phosphomethylpyrimidine synthase | Fe | -0.56 | 4.0E-02 | -0.84 | 2.5E-03 |
| PA5025 | MetY | Homocysteine synthase | | 0.86 | 9.1E-03 | | |
| **Respiration** | | | | | | | |
| PA0527 | Dnr | Transcriptional regulator | | -0.94 | 1.5E-02 | | |
| PA1173 | NapB | Periplasmic nitrate reductase | Fe | -0.99 | 7.1E-03 | | |
| PA1174 | NapA | Periplasmic nitrate reductase | Fe | -1.31 | 1.2E-12 | -1.58 | 2.5E-04 |
| PA1175 | NapD | NapA signal peptide-binding chaperone | | -0.72 | 1.4E-02 | | |
| PA1176 | NapF | Ferredoxin-type protein | Fe | -0.88 | 2.0E-02 | | |
| PA1544 | Anr | Transcriptional activator protein | Fe | 0.69 | 3.4E-05 | | |
| PA2266 | | Probable cytochrome c | Fe | -0.56 | 4.6E-01 | | |
| PA3331 | | Cytochrome P450 | Fe | -0.67 | 7.6E-04 | -2.73 | 1.9E-02 |
| PA3872 | NarI | Respiratory nitrate reductase gamma chain | Fe | -0.58 | 3.6E-02 | | |
| PA4133 | CcoN | Cytochrome c oxidase subunit (Cbb3-type) | Fe | -1.11 | 1.2E-02 | -2.63 | 1.9E-03 |

| | | | | | | | |
|---|---|---|---|---|---|---|---|
| **Anr regulon** | | | | | | | |
| PA1546 | HemN | Oxygen-independent coproporphyrinogen III oxidase (CPO) | Fe | -1.63 | 2.6E-06 | -1.33 | 4.8E-04 |
| PA1673 | | Bacteriohemerythrin | Fe | -0.95 | 5.3E-06 | -2.13 | 1.1E-03 |
| PA3049 | Rmf | Ribosome modulation factor | | 0.74 | 3.8E-03 | 1.42 | 3.3E-03 |
| PA3126 | IbpA | Heat-shock protein IbpA | | -0.60 | 2.5E-04 | | |
| PA3572 | | Hypothetical protein | | -0.52 | 2.5E-03 | | |
| PA3919 | YlaK | PINc domain-containing protein | | -0.77 | 5.5E-02 | | |
| PA4587 | CcpR | Cytochrome c551 peroxidase | Fe | -0.52 | 1.3E-04 | | |
| PA4880 | | Probable bacterioferritin | Fe | -2.95 | 8.1E-16 | -3.54 | 4.5E-06 |
| PA5475 | | Acetyltransferase | | -0.61 | 4.1E-02 | -2.96 | 2.3E-04 |
| **Oxidative stress regulation** | | | | | | | |
| PA0140 | AhpF | Alkyl hydroperoxide reductase subunit F | | -0.60 | 2.1E-06 | | |
| PA0849 | TrxB2 | Thioredoxin reductase | | -0.77 | 4.2E-04 | | |
| PA2185 | KatN | Non-heme catalase KatN | Mn | -0.91 | 1.2E-01 | | |
| PA2580 | MdaB | Flavodoxin_2 domain-containing protein | | -0.55 | 1.0E-02 | | |
| PA3450 | LsfA | Probable antioxidant protein | | 2.49 | 1.2E-21 | | |
| PA3533 | GrxD | Glutaredoxin | | -0.81 | 3.6E-08 | -3.28 | 2.9E-04 |
| PA4236 | KatA | Catalase | Fe | -1.08 | 1.4E-08 | -3.06 | 7.4E-06 |
| PA4366 | SodB | Superoxide dismutase [Fe] | Fe | -0.96 | 7.1E-03 | -4.28 | 4.2E-06 |
| PA4468 | SodA | Superoxide dismutase [Mn] | Mn | 2.81 | 2.6E-14 | 3.91 | 4.4E-05 |
| **Nucleotide synthesis and metabolism** | | | | | | | |
| PA0441 | Dht | D-hydantoinase/ dihydropyrimidinase (DHPase) | Zn | -0.71 | 2.2E-02 | | |
| PA1155 | NrdB | Ribonucleoside-diphosphate reductase subunit beta | Fe | -0.61 | 2.0E-02 | | |
| PA1156 | NrdA | Ribonucleoside-diphosphate reductase | | -0.62 | 1.9E-06 | | |
| PA1932 | | Probable hydroxylase molybdopterin-containing subunit | | -0.89 | 9.0E-02 | | |
| **Lipid metabolism** | | | | | | | |
| PA0347 | GlpQ | Glycerophosphoryl diester phosphodiesterase, periplasmic | | -1.06 | 2.6E-11 | | |
| PA2862 | LipA | Triacylglycerol lipase | | -0.52 | 2.1E-01 | | |
| PA3092 | FadH1 | 2,4-dienoyl-CoA reductase FadH1 | | -1.11 | 2.0E-04 | | |
| PA3319 | PlcN | Non-hemolytic phospholipase C (PLC-N) | | -0.84 | 3.6E-14 | | |
| PA3333 | FabH | 3-oxoacyl-[acyl-carrier-protein] synthase 3 | | -0.85 | 4.6E-06 | -1.67 | 6.7E-04 |
| PA4350 | OlsB | Uncharacterized protein | | -0.55 | 5.0E-02 | | |
| PA4353 | YajB | Uncharacterized protein | | -0.56 | 6.0E-03 | | |
| PA4661 | PagL | Lipid A deacylase PagL | | 0.51 | 6.1E-02 | | |
| **Secreted proteins and secretion systems** | | | | | | | |
| PA0572 | ImpA | Immunomodulating metalloprotease | Zn | 0.71 | 1.1E-02 | | |
| PA0688 | PhoA | Alkaline phosphatase L (L-AP) | | -0.74 | 5.8E-09 | | |
| PA1245 | AprX | Uncharacterized protein | | 3.02 | 3.9E-10 | 3.29 | 1.1E-01 |

| | | | | | | | |
|---|---|---|---|---|---|---|---|
| PA1246 | AprD | Alkaline protease secretion ATP-binding protein AprD | | 2.25 | 2.2E-09 | 3.43 | 5.1E-04 |
| PA1247 | AprE | Alkaline protease secretion protein AprE | | 2.11 | 3.1E-03 | -0.17 | 9.0E-02 |
| PA1249 | AprA | Serralysin (Alkaline metalloproteinase) (AP) | Zn | 1.82 | 1.4E-10 | 0.83 | 1.0E-02 |
| PA1250 | AprI | Proteinase inhibitor (Aprin) | Zn | 0.96 | 1.0E-03 | | |
| PA3099 | XcpV | Type II secretion system protein I | | 0.79 | 1.4E-01 | | |
| **Oxidation-reduction processes** | | | | | | | |
| PA0785 | AzoR1 | FMN-dependent NADH-azoreductase 1 | | -0.69 | 2.3E-01 | | |
| PA0840 | | Probable oxidoreductase | | -0.51 | 1.3E-02 | | |
| PA2033 | | FAD-binding FR-type domain-containing protein | | 1.72 | 5.8E-10 | 2.42 | 4.1E-05 |
| PA2158 | | Probable alcohol dehydrogenase (Zn-dependent) | Zn | 0.75 | 1.4E-01 | | |
| PA2378 | | Probable aldehyde dehydrogenase | | -0.93 | 8.1E-05 | -1.64 | 2.7E-03 |
| PA2379 | | Probable oxidoreductase | Fe | -1.03 | 1.4E-03 | -2.15 | 2.5E-03 |
| PA5150 | | Probable short-chain dehydrogenase | | 1.45 | 1.0E-09 | 2.53 | 7.4E-05 |
| PA5187 | | Probable acyl-CoA dehydrogenase | | -0.61 | 2.8E-01 | | |
| PA5188 | | Probable 3-hydroxyacyl-CoA dehydrogenase | | -0.63 | 1.4E-02 | -2.62 | 5.8E-05 |
| **Other cellular transporters** | | | | | | | |
| PA2203 | | Probable amino acid permease | | 1.74 | 6.8E-03 | | |
| PA2491 | MexS | Probable oxidoreductase | | -1.06 | 1.2E-09 | -1.05 | 3.0E-05 |
| PA2493 | MexE | RND multidrug efflux membrane fusion protein | | -0.81 | 2.3E-07 | | |
| PA2494 | MexF | Efflux pump membrane transporter | | -0.75 | 3.2E-07 | | |
| PA3280 | OprO | Porin O | | -1.02 | 8.4E-07 | | |
| PA3931 | | Uncharacterized protein | | 1.54 | 1.8E-22 | | |
| PA5103 | PuuR | OpuAC domain-containing protein | | 0.71 | 1.2E-01 | | |
| PA5217 | | Probable binding protein component of ABC iron transporter | | 0.50 | 8.7E-02 | 1.86 | 2.3E-05 |
| **Others** | | | | | | | |
| PA0168 | YigZ | Uncharacterized protein | | 0.56 | 2.9E-01 | | |
| PA0225 | | Probable transcriptional regulator | | 0.78 | 1.0E-01 | | |
| PA0277 | | Peptidase_M48 domain-containing protein | | -0.58 | 3.6E-02 | | |
| PA0284 | | Uncharacterized protein | | 2.35 | 3.5E-07 | -2.90 | 1.9E-03 |
| PA0312 | | DUF523 domain-containing protein | | -1.44 | 3.1E-02 | -1.63 | 1.3E-03 |
| PA0321 | AphB | Acetylpolyamine amidohydrolase 2 | | -0.53 | 1.7E-03 | | |
| PA0423 | PasP | UPF0312 protein PA0423 | | 0.61 | 3.8E-06 | 1.21 | 3.4E-04 |
| PA0540 | | Uncharacterized protein | | 0.82 | 6.5E-03 | | |
| PA0622 | | Probable bacteriophage protein | | 0.62 | 1.3E-03 | | |
| PA0623 | | Probable bacteriophage protein | | 0.68 | 1.0E-02 | | |
| PA0633 | | Uncharacterized protein | | 1.19 | 3.0E-02 | | |
| PA0634 | | Uncharacterized protein | | 0.52 | 2.8E-02 | | |
| PA0641 | | Probable bacteriophage protein | | 0.96 | 4.1E-02 | | |
| PA0757 | | Probable two-component sensor | | 0.58 | 5.6E-01 | | |
| PA0807 | AmpDh3 | AmpDh3 | | 0.51 | 1.6E-03 | | |

| | | | | | | | |
|---|---|---|---|---|---|---|---|
| PA0929 | PirR | Two-component response regulator | | -1.24 | 6.0E-05 | ND | |
| PA0981 | | Uncharacterized protein | | -0.97 | 9.2E-05 | | |
| PA0984 | | Colicin immunity protein | | 0.52 | 1.0E-01 | | |
| PA0985 | PyoS5 | Pyocin S5 | | 0.91 | 6.7E-07 | | |
| PA1017 | PauA | Pimeloyl-CoA synthetase | | 1.23 | 5.2E-01 | | |
| PA1026 | | Uncharacterized protein | | -0.80 | 4.2E-02 | | |
| PA1484 | | Probable transcriptional regulator | | 0.62 | 1.3E-01 | | |
| PA1522 | | Uncharacterized protein | | -0.55 | 1.5E-01 | | |
| PA1727 | MucR | Uncharacterized signaling protein PA1727 | | -0.71 | 5.7E-02 | | |
| PA1784 | | Alginate_lyase2 domain-containing protein | | 1.36 | 1.9E-02 | | |
| PA1837 | | Uncharacterized protein | | 1.10 | 6.2E-02 | -3.18 | 1.7E-04 |
| PA2034 | | Methyltransf_25 domain-containing protein | | 2.07 | 1.3E-09 | 2.45 | 1.1E-04 |
| PA2048 | | ABM domain-containing protein | | -0.71 | 1.0E-03 | | |
| PA2066 | | DUF218 domain-containing protein | | 0.99 | 3.2E-02 | | |
| PA2086 | | Probable epoxide hydrolase | | 3.48 | 1.6E-17 | | |
| PA2161 | | Uncharacterized protein | | 0.62 | 3.7E-01 | 3.92 | 1.1E-04 |
| PA2171 | | Hemerythrin domain-containing protein | | -2.46 | 1.4E-01 | -1.93 | 3.4E-03 |
| PA2184 | YciE | DUF892 domain-containing protein | | 0.63 | 1.3E-03 | | |
| PA2204 | | Probable binding protein component of ABC transporter | | 1.82 | 4.9E-23 | | |
| PA2309 | | Uncharacterized protein | | 1.50 | 9.9E-04 | | |
| PA2328 | | Uncharacterized protein | | 1.15 | 1.8E-05 | | |
| PA2384 | | Uncharacterized protein | | 2.81 | 2.6E-12 | 3.59 | 8.3E-05 |
| PA2414 | | L-sorbosone dehydrogenase | | 0.75 | 1.1E-01 | | |
| PA2448 | | Amidohydro_3 domain-containing protein | | 0.60 | 2.6E-04 | | |
| PA2531 | | Probable aminotransferase | | 2.63 | 1.7E-07 | 1.57 | 5.1E-05 |
| PA2535 | | Probable oxidoreductase | | -0.53 | 3.6E-08 | | |
| PA2599 | | PBPb domain-containing protein | | 0.79 | 2.7E-02 | | |
| PA2603 | | Probable thiosulfate sulfurtransferase | | 0.58 | 2.5E-02 | | |
| PA2635 | | Uncharacterized protein | | -1.18 | 2.3E-04 | | |
| PA2765 | | Uncharacterized protein | Fe | -0.92 | 3.2E-09 | -1.20 | 8.3E-03 |
| PA2786 | | GAF domain-containing protein | | 3.91 | 1.2E-17 | | |
| PA2804 | | HD_domain domain-containing protein | | -1.16 | 1.1E-05 | | |
| PA2821 | | Probable glutathione S-transferase | | -1.06 | 4.5E-07 | | |
| PA2831 | | Uncharacterized protein | Zn | -0.57 | 3.7E-08 | -1.68 | 6.4E-04 |
| PA2915 | | Lactamase_B domain-containing protein | | -0.64 | 1.2E-02 | | |
| PA2956 | | Elp3 domain-containing protein | Fe | -0.97 | 7.2E-02 | -2.56 | 6.8E-04 |
| PA3009 | | Uncharacterized protein | | -0.76 | 8.7E-02 | -3.55 | 2.2E-02 |
| PA3010 | | Uncharacterized protein | | -0.54 | 8.3E-03 | | |
| PA3022 | | MOSC domain-containing protein | Mo | -0.63 | 6.0E-03 | -1.50 | 1.1E-03 |
| PA3067 | | Probable transcriptional regulator | | -1.20 | 3.2E-01 | | |
| PA3124 | | Probable transcriptional regulator | | -1.20 | 6.7E-02 | | |
| PA3219 | | Metallophos_2 domain-containing protein | | -0.98 | 2.0E-08 | | |
| PA3240 | | Putative quercetin 2,3-dioxygenase PA3240 (Putative quercetinase) | | -0.63 | 1.0E-01 | | |
| PA3277 | | Probable short-chain dehydrogenase | | 0.57 | 2.4E-01 | | |

| | | | | | | | |
|---|---|---|---|---|---|---|---|
| PA3321 | | Probable transcriptional regulator | | -0.82 | 4.2E-02 | | |
| PA3327 | | Probable non-ribosomal peptide synthetase | | -1.48 | 2.3E-04 | | |
| PA3328 | | Probable FAD-dependent monooxygenase | | -1.01 | 1.1E-02 | | |
| PA3329 | | Condensation domain-containing protein | | -0.79 | 6.8E-09 | | |
| PA3330 | | Probable short chain dehydrogenase | | -1.20 | 1.8E-05 | | |
| PA3332 | | Uncharacterized PhzA/B-like protein PA3332 | | -0.64 | 2.4E-02 | -1.04 | 1.9E-04 |
| PA3441 | SsuF | Probable molybdopterin-binding protein | | 3.04 | 3.2E-09 | | |
| PA3815 | IscR | HTH-type transcriptional regulator IscR | | -0.56 | 8.9E-02 | 1.04 | 2.6E-02 |
| PA3844 | | GST N-terminal domain-containing protein | | -0.88 | 6.7E-09 | | |
| PA3846 | | Isochorismatase domain-containing protein | | -0.95 | 3.9E-03 | -1.09 | 7.2E-03 |
| PA3865.1 | | Pyosin S4 immunity protein | | 2.14 | 2.8E-02 | | |
| PA3866 | | Pyocin protein | | 1.67 | 2.2E-12 | 2.08 | 1.4E-04 |
| PA3932 | | Probable transcriptional regulator | | 2.24 | 6.1E-07 | | |
| PA4131 | | Probable iron-sulfur protein | Fe | -1.29 | 3.8E-03 | -2.62 | 2.8E-06 |
| PA4165 | | Probable transcriptional regulator | | 0.60 | 3.0E-02 | | |
| PA4195 | | Probable binding protein component of ABC transporter | | 2.01 | 2.0E-03 | -2.29 | 6.4E-03 |
| PA4197 | BfiS | | | -1.20 | 1.8E-01 | | |
| PA4325 | | DUF4124 domain-containing protein | | -0.87 | 2.7E-01 | | |
| PA4370 | IcmP | Insulin-cleaving metalloproteinase outer membrane protein | | 1.12 | 1.1E-14 | 1.85 | 1.2E-03 |
| PA4371 | | Uncharacterized protein | Fe | 0.64 | 2.8E-08 | | |
| PA4373 | | Uncharacterized protein | | 0.73 | 2.4E-01 | | |
| PA4384 | | Uncharacterized protein | | 0.50 | 1.6E-01 | | |
| PA4390 | | Uncharacterized protein | | 1.05 | 1.3E-01 | 1.12 | 3.0E-04 |
| PA4573 | | Uncharacterized protein | | 1.95 | 1.9E-01 | | |
| PA4664 | PrmC | S-adenosylmethionine-dependent methyltransferase | | -0.75 | 1.2E-02 | | |
| PA4674 | VapI | Antotoxin HigA | | -0.64 | 1.6E-01 | | |
| PA4692 | MsrP | Protein-methionine-sulfoxide reductase catalytic subunit MsrP | | -0.71 | 1.1E-01 | | |
| PA4704 | CbpA | cAMP-binding protein A | | -0.55 | 2.9E-01 | | |
| PA4728 | FolK | 2-amino-4-hydroxy-6-hydroxymethyldihydropteridine pyrophosphokinase | | 0.96 | 5.4E-02 | | |
| PA4792 | | GP-PDE domain-containing protein | | 1.61 | 1.2E-11 | | |
| PA4875 | | MOSC domain-containing protein | Mo | -1.06 | 1.7E-04 | | |
| PA4928 | YgiR | UPF0313 protein | Fe | -1.31 | 6.7E-03 | | |
| PA4943 | HflX | GTPase HflX (GTP-binding protein HflX) | | -0.55 | 3.3E-01 | | |
| PA5081 | | Nudix hydrolase domain-containing protein | | 0.69 | 3.1E-02 | | |
| PA5189 | | Probable transcriptional regulator | | 0.50 | 4.4E-01 | | |
| PA5506 | | Uncharacterized protein | | -0.66 | 2.2E-01 | | |

**Table S3**: Resonance assignments for $^{1}$H and $^{13}$C chemical shifts of amino acids and other metabolites identified by NMR spectroscopy in the wt and Δ*bfd* cells.

| Metabolite | Structure | Group | $\delta_H$ (ppm) | $\delta_C$ (ppm) | [1]H Multiplicity |
|---|---|---|---|---|---|
| **Alanine** | 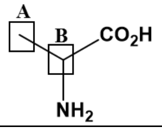 | **A**-$CH_3$<br>**B**-CH | 1.471<br>3.771 | 18.83<br>53.29 | *d*<br>*q* |
| **Cystine** | 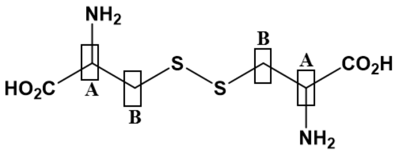 | **A**-CH<br>**B**-$CH_2$ | 4.103<br>3.180, 3.378 | 56.14<br>40.55 | *dd*<br>*dd* |
| **Glycine** | 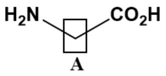 | **A**-$CH_2$ | 3.545 | 44.13 | *s* |
| **Leucine** | 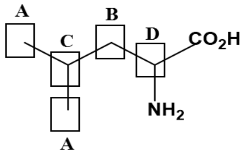 | **A**-$CH_3$<br>**B**-$CH_2$<br>**C**-CH<br>**D**-CH | 0.949<br>1.701<br>1.701<br>3.719 | 24.75, 23.59<br>42.53<br>26.87<br>56.11 | *t*<br>*m*<br>*m*<br>*m* |
| **Lysine** | 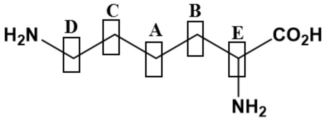 | **A**-$CH_2$<br>**B**-$CH_2$<br>**C**-$CH_2$<br>**D**-$CH_2$<br>**E**-CH | 1.465<br>1.716<br>1.895<br>3.012<br>3.745 | 24.15<br>29.14<br>32.63<br>41.75<br>57.19 | *m*<br>*quint*<br>*m*<br>*t*<br>*t* |
| **Methionine** | 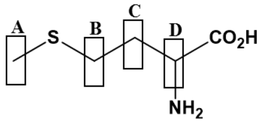 | **A**-$CH_3$<br>**B**-$CH_2$<br>**C**-$CH_2$<br>**D**-CH | 2.122<br>2.629<br>2.183, 2.122<br>3.850 | 16.64<br>31.51<br>32.40<br>56.58 | *m*<br>*t*<br>*m*<br>*dd* |
| **Valine** | 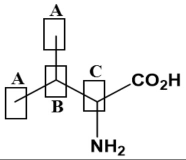 | **A**-$CH_3$<br>**B**-CH<br>**C**-CH | 0.981, 1.034<br>2.266<br>3.599 | 19.37, 20.70<br>31.83<br>63.08 | *d*<br>*m*<br>*d* |
| **Oxidized glutathione** | 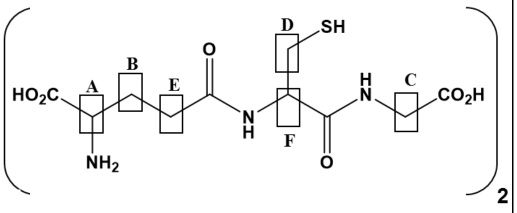 | **A**-$CH_2$<br>**B**-$CH_2$<br>**C**-$CH_2$<br>**D**-$CH_2$<br>**E**-CH<br>**F**-CH | 2.16<br>2.53<br>3.77<br>3.14<br>3.77<br>4.75 | 28.98<br>34.10<br>46.18<br>41.39<br>56.80<br>55.30 | *m*<br>*m*<br>*m*<br>*dd*<br>*m*<br>*q* |
| **Glycine-betaine** | 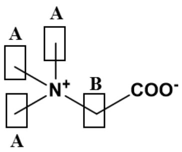 | **A**-$CH_3$<br>**B**-$CH_2$ | 3.255<br>3.895 | 56.08<br>68.86 | *s*<br>*s* |

Chemical shift assignments were made in reference to the publicly accessible Human Metabolome and BioMagResBank databases [5,6]. Assignments were corroborated with the aid of $^{1}$H-$^{13}$C-HSQC and TOCSY spectra, as presented in Materials and Methods.

**References**


1. Kanehisa, M.; Goto, S.; Sato, Y.; Kawashima, M.; Furumichi, M.; Tanabe, M. Data, information, knowledge and principle: back to metabolism in KEGG. *Nucleic acids research* **2014**, *42*, D199-D205, doi:10.1093/nar/gkt1076.
2. Winsor, G.L.; Griffiths, E.J.; Lo, R.; Dhillon, B.K.; Shay, J.A.; Brinkman, F.S. Enhanced annotations and features for comparing thousands of Pseudomonas genomes in the Pseudomonas genome database. *Nucleic Acids Res* **2016**, *44*, D646-653, doi:10.1093/nar/gkv1227.
3. Apweiler, R.; Bairoch, A.; Wu, C.H.; Barker, W.C.; Boeckmann, B.; Ferro, S.; Gasteiger, E.; Huang, H.; Lopez, R.; Magrane, M., et al. UniProt: the Universal Protein knowledgebase. *Nucleic Acids Res* **2004**, *32*, D115-119, doi:10.1093/nar/gkh131.
4. Nelson, C.E.; Huang, W.; Brewer, L.K.; Nguyen, A.T.; Kane, M.A.; Wilks, A.; Oglesby-Sherrouse, A.G. Proteomic Analysis of the Pseudomonas aeruginosa Iron Starvation Response Reveals PrrF Small Regulatory RNA-Dependent Iron Regulation of Twitching Motility, Amino Acid Metabolism, and Zinc Homeostasis Proteins. *J Bacteriol* **2019**, *201*, doi:10.1128/JB.00754-18.
5. Wishart, D.S.; Feunang, Y.D.; Marcu, A.; Guo, A.C.; Liang, K.; Vazquez-Fresno, R.; Sajed, T.; Johnson, D.; Li, C.; Karu, N., et al. HMDB 4.0: the human metabolome database for 2018. *Nucleic Acids Res* **2018**, *46*, D608-D617, doi:10.1093/nar/gkx1089.
6. Ulrich, E.L.; Akutsu, H.; Doreleijers, J.F.; Harano, Y.; Ioannidis, Y.E.; Lin, J.; Livny, M.; Mading, S.; Maziuk, D.; Miller, Z., et al. BioMagResBank. *Nucleic Acids Res* **2008**, *36*, D402-408, doi:10.1093/nar/gkm957.
